# Weighted–VAE: A deep learning approach for multimodal data generation applied to experimental *T. cruzi* infection

**DOI:** 10.1371/journal.pone.0315843

**Published:** 2025-03-24

**Authors:** Blanca Vazquez, Nidiyare Hevia-Montiel, Jorge Perez-Gonzalez, Paulina Haro

**Affiliations:** 1 Unidad Académica del Instituto de Investigaciones en Matemáticas Aplicadas y en Sistemas del Estado de Yucatán, Universidad Nacional Autónoma de México, Mérida, Yucatán, Mexico; 2 Instituto de Investigaciones en Ciencias Veterinarias, Universidad Autónoma de Baja California, Mexicali, Baja California, Mexico; University of Southern California, UNITED STATES OF AMERICA

## Abstract

Chagas disease (CD), caused by the protozoan parasite *Trypanosoma cruzi (T. cruzi)*, represents a major public health concern in most of the American continent and causes 12,000 deaths every year. CD clinically manifests in two phases (acute and chronic), and the diagnosis can result in complications due to the difference between phases and the long period between them. Still, strategies are lacking for the automatic diagnosis of healthy and *T. cruzi*-infected individuals with missing and limited data. In this work, we propose a Weighted Variational Auto-Encoder (W–VAE) for imputing and augmenting multimodal data to classify healthy individuals and individuals in the acute or chronic phases of *T. cruzi* infection from a murine model. W–VAE is a deep generative architecture trained with a new proposed loss function to which we added a weighting factor and a masking mechanism to improve the quality of the data generated. We imputed and augmented data using four modalities: electrocardiography signals, echocardiography images, Doppler spectrum, and ELISA antibody titers. We evaluated the generated data through different multi-classification tasks to identify healthy individuals and individuals in the acute or chronic phase of infection. In each multi-classification task, we assessed several classifiers, missing rates, and feature-selection methods. The best obtained accuracy was 92 ± 4*%* in training and 95% in the final test using a Gaussian Process Classifier with a missing rate of 50%. The accuracy achieved was 95% for individuals in healthy and acute phase and 100% for individuals in the chronic phase. Our approach can be useful in generating data to study the phases of *T. cruzi* infection.

## Introduction

*Trypanosoma cruzi* (*T. cruzi*) is a parasitic protozoan that causes Chagas disease (CD) or American trypanosomiasis [1]. The World Health Organization (WHO) estimated that 6 to 7 million people worldwide are infected with *T. cruzi* in their reported of 2023 [2]. CD is endemic in 21 countries in Latin America and is predominantly transmitted by triatomine insects, which deposit their infected feces in skin wounds or on mucous membranes. Other transmission modalities are blood transfusion, organ transplant, mother-to-child, exposure in the lab, and eating uncooked food contaminated with feces from infected insects [3,4].

CD clinically manifests in two phases: acute and chronic. During the acute phase, parasites may be found in the circulating blood. This phase of infection is usually mild or asymptomatic, but the most common symptoms reported are fever, inflammation at the inoculation site, rash, and headache [5,6]. During the chronic phase, on the other hand, few or no parasites are found in the blood. Still, it is estimated that 30–40% of infected patients can develop complications (mainly cardiomyopathy or megaviscera in the form of megaesophagus, megacolon, or both) [3]. According to Feldman and McNamara, CD is the most common cause of infectious myocarditis globally [1].

The diagnosis of CD and the identification of its clinical phase represents a challenge because diagnosis depends mainly on the patient’s current phase [7]. For instance, in the acute phase, diagnosis is based on parasitological tests to determine whether parasites are present in the blood, while, in the chronic phase, diagnosis involves immunoserological methods, which detect antibodies that are created against the infection. The most common immunoserological method is Enzyme Linked Immunosorbent Assay (ELISA) [8]. Unfortunately, some patients develop heart disease and myocardium damage; hence, tools like echocardiography (ECHO), electrocardiography (ECG), and spectral ultrasound Doppler (DOPPLER) can provide useful diagnostic and prognostic information in the management of patients with CD [9].

The relevance of all these modalities (ELISA, ECHO, ECG, and DOPPLER) is that they assist in the diagnosis of CD. Automatic diagnosis and distinction of healthy individuals and infection phases using these modalities, however, have been little explored using missing and limited data. To address these limitations, we propose a new multi-classification methodology to detect healthy individuals and individuals in the acute and chronic phases of *T. cruzi* infection based on a new proposed variational auto-encoder (VAE) architecture for imputing and augmenting data applied to a murine model.

## Related works

### Machine learning in *T. cruzi* detection

The automatic identification of healthy individuals and infected individuals with *T. cruzi* has been studied through the analysis of one or more multimodal data points. For instance, Hevia et al. [10] presented a binary classification approach to identify healthy individuals vs. infected individuals using the ELISA, ECG, ECHO, and DOPPLER modalities from a murine model. They found that classification algorithms fed with different multimodal data showed good performance in the discrimination of individuals infected. Similarly, Haro et al. [11] evaluated two approaches of classification: i) binary (healthy individuals vs. infected individuals) and ii) multiclass (healthy vs. acute phase vs. chronic phase) using the ECG modality in a murine model. They achieved an accuracy of 91.3% in the multi-classification and concluded that it is possible to discriminate between healthy individuals and individuals in the acute phase.

Valdez et al. [12] explored a multi-classification of healthy individuals, individuals with Chagas without cardiac involvement, and individuals with Chagas with mild to moderate incipient heart failure using the ECG modality. They used PCA as their feature-selection method and a densely connected neural network as the classifier. Added Gaussian noise to augment the data achieved a total accuracy of 98%. The authors found that feature extraction and data augmentation improved network performance by overcoming the data limitations. Similarly, Vizcardo et al. [13] evaluated a multi-classification of healthy individuals, individuals with Chagas without ECG alterations, and individuals with Chagas with ECG alterations. They computed an orthogonal base to discriminate among the individuals and achieved a sensitivity of over 60% to classify healthy individuals vs. individuals with Chagas. The authors concluded that the use of non-linear modeling techniques can detect abnormalities early in CD diagnosis.

Although these works present interesting results, they have some limitations: i) they did not address the distinction multiclass of healthy individuals or individuals in the acute or chronic stages of infection using different multimodal data and ii) they did not attend the issues of missing or scarce data.

Several additional works have studied the identification of *T. cruzi* infection by identifying trypomastigote nests in images. Morais et al. [14] used a Random Forest classifier to detect and count *T. cruzi* trypomastigotes in blood-smear samples acquired by a mobile device camera. They extracted a set of morphometric, color, and texture features, which they used to train their classifier. The authors concluded that automating image analysis acquired with a mobile device is a viable for reducing costs and gaining efficiency in the use of the optical microscope. Similarly, Neto [15] presented a K-nearest neighbors (KNN) classifier to find trypomastigotes in blood-smear samples using a mobile device to extract the geometry, color, and texture features. Neto noted the importance of developing computer-vision approaches as alternatives to detect trypanosomes efficiently. Cetina et al. [16] provided a AdaBoost classifier to detect trypomastigotes in blood images using Gaussian discriminant analysis. For each sample, they extracted features of shape and color to detect the parasite, demonstrating that Chagas parasites can be identified automatically using machine learning methods with high accuracy.

Ojeda et al. [17] implemented a convolutional neural network based on the U-Net architecture for segmenting the *T. cruzi* parasite on blood-sample images. They tested the Weighted Binary Cross-Entropy and Binary Cross-Entropy loss functions to train their architecture. The authors mentioned that a proper segmentation can help experts obtain a quick diagnosis of CD. Similar work was described by Hevia et al. [18], who presented an automatic segmentation approach of amastigote nets in histopathology images based on the U-Net architecture in combination with data-augmentation approaches. They concluded that adequate nest segmentation and quantification could help the study of CD in different clinical centers. In all these works based on images, the authors concluded that the creation of an architecture or prototype based on deep learning could be useful to automatically detect Chagas parasites and accelerate the diagnosis of the disease. They did not, however, distinguish healthy individuals, or individuals in acute and chronic stages of infection, where this identification could be useful to help experts with CD study, follow-up, and analysis.

Missing and scarce data are common problems that lead to biased estimates and invalid conclusions in biomedicine research [19,20]. The missing-data problem is common in all domains and is caused by data-processing errors, incomplete data entry, equipment malfunctions, and so on [21].The scarce-data problem results from data that include sensitive information and cannot be freely shared [22]. Moreover, accessing substantial amounts of data can be time-consuming, costly, and challenging [23].

The problem of missing data has been attended through imputation techniques, such as replacement with statistical values or using machine learning models. Multivariate imputation by chained equations (MICE), K-nearest neighbor (KNN), and simple imputation are techniques frequently used to address the problem of missing data [24–26]. In particular, the MICE method estimates data through an iterative process; in each iteration, each feature is imputed using the values from all the features in the dataset [25,27]. By contrast, in KNN, each sample with missing values is imputed using the mean value from the K-nearest neighbors found in the dataset [28]. In simple imputation, each missing value is replaced by a descriptive statistic (mean, median, or most frequent) alongside each feature [27].

On the other hand, some studies have used the Synthetic Minority Oversampling Technique (SMOTE) to augment data and address the scarce-data problem [29,30]. This algorithm randomly selects an instance from the minority class, then randomly selects an instance from the K-nearest neighbor of that instance, and finally generates a new instance based on interpolation between the two [31]. Some studies, however, have mentioned that the augmented data produced by SMOTE may not precisely match the original distribution of the class [30,32].

Recently, deep learning architectures have been used to impute and augment data, outperforming other existing methods. Some advantages of these architectures are: i) they capture the complex structure of high-dimensional data, ii) they model non-linear associations, and iii) they avoid making strong assumptions about the data distribution [33–35]. VAEs’ main advantage is that they are deep learning architectures able to learn the data distribution to generate new meaningful data from two neural networks, called the *encoder* and *decoder*. VAEs have been used in multiple applications in biomedicine research, such as biological sequence analyses, medical image classification, anomaly detection, among others [35,36].

### Deep learning for imputing and augmenting data

VAEs are generative architectures that has been used as data-imputation techniques and to address the scarce-data problem. For instance, Lina et al. [37] used a VAE for genomic missing-value imputation and demonstrated its effectiveness in transcriptome and methylome data analysis. They found that VAEs are an alternative to traditional methods for data imputation, especially in the setting of large-scale data and certain missing-not-at-random scenarios.

Similarly, Huang et al. [38] proposed a modified VAE to impute carotid artery lesion data which are using for the early identification of abnormal arteries. The authors noted that the imputed data by the proposed VAE achieved a superior performance in the classification of abnormal arteries compared with other imputations methods, such as KNN or filling data with the mean. Pereira et al. [39] presented an architecture based on VAE to impute data from 34 public datasets based on routinely collected healthcare data. The authors evaluated data with missing rates of between 10% and 80%. They reported that their proposed VAE significantly outperformed state-of the-art imputation strategies, such as MICE, KNN, and simple imputation.

As a data-augmentation techniques, the use of VAE is crucial because they increase the size and diversity of multimodal data [23]. Rajaram et al. [40] presented a novel framework to conduct data augmentation on multimodal data for cancer-survival prediction. They concluded that using VAE as an augmentation method provides significant performance improvements over existing state-of-the-art methods. In the same way, Saldanha et el. [41] described a methodology to augment data based on VAEs for improvement of respiratory disease classification; they synthesized respiratory sounds of various categories and compared the influence of increasing data on the performance of various lung-sound classifiers. They observed a significant improvement in the classification performance metrics on augmented datasets.

In these works based on VAEs, the authors remarked on the advantages of use this deep learning architecture to solve the problems of missing data or limited data. There is, however, a lack of strategies that attend these problems in the detection of stages of *T. cruzi* infection. According to [42], the lack of awareness and understanding of the disease among both physicians and populations at risk increasing the complexity of case registration. Therefore, having access to large databases with complete data is a challenge [43].

The present study aims to introduce a new multi-classification methodology to identify healthy individuals, and individuals in the acute and chronic phases of *T. cruzi* infection by imputing and augmenting data using a W–VAE proposal. Our proposal could be useful in solving the problems of missing and limited data. The major contributions of the paper are:

- We present a Weighted VAE–based approach (W–VAE) for imputing and augmenting multimodal data for the multi-classification of *T. cruzi* from a murine model.
- We implement a new loss function, adding a weighing factor and masking mechanism to improve the performance of the quality of the data generated by the proposed W–VAE.
- We evaluate the quality of the generated data through different multi-classification tasks. We confirm that the proposed W–VAE can generate multimodal data with performances very close to the real multimodal data; in fact, even better performances were obtained with high missing rates.
- We propose new strategies for classifying healthy individuals and individuals in the acute or chronic phase of infection in an experimental model from the multimodal data.

The paper is structured as follows. The Materials and Methods section introduces details of the proposed W–VAE to impute and augment the multimodal data. It also describes the process of classifying healthy individuals, acute-phase individuals, and chronic-phase individuals. The Results section reports the experimental results. The Discussion section analyzes the results and presents the limitations of the present work. Finally, the Conclusion section closes the paper by providing some remarks on its significance.

## Materials and methods

**Fig 1:**
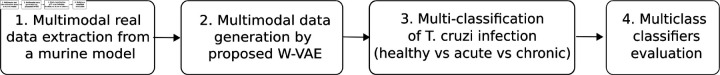
Overview of the proposal methodology for the multi-classification of healthy individuals and individuals in the acute or chronic phase of *T. cruzi* infection using a deep generative architecture (W–VAE).

We propose a new multi-classification methodology to identify healthy individuals and individuals in the acute or chronic phases of *T. cruzi* infection using a proposed W–VAE, as shown in Fig 1. Our methodology consists of four major steps: 1) extracting the multimodal data of healthy individuals and infected individuals with *T. cruzi* from a murine model, 2) imputing and augmenting the multimodal data from a proposed W–VAE, 3) classifying healthy individuals, acute-phase individuals, and chronic-phase individuals with *T. cruzi* infection, and 4) evaluating the performance of the classifiers. The details are described in the subsections below.

### Murine model of *T. cruzi* infection

For the experimental study, 193 healthy female ICR mice were employed to study the acute and chronic phases of *T. cruzi* infection. The duration of the experimental murine model was 35 days for the acute phase and 120 days for the chronic phase. At the beginning of the experiment, the mice were between 6 and 8 weeks old. All animals used in the experiment were handled according to the Care and Use of Laboratory Animals Guide (8th edition). The protocol was approved by the Ethics Committee of the Centro de Investigaciones Regionales Dr. Hideyo Noguchi (CIRB-006-2017) at the Universidad Autónoma de Yucatán, México.

The acute-phase experiment employed 108 mice. For the infected group, 66 mice were inoculated with 1000 blood trypomastigotes of strain H1 (TcI lineage) *T. cruzi* via intraperitoneal (IP). The rate of mortality was 24%, and the causes of death were humanitarian endpoint due to respiratory distress, dehydration, hypothermia, and sudden death due to cardiac failure. For the healthy control group, 42 mice were given a physiological saline solution IP.

For the experimental chronic-phase infection, 85 mice were used. For the infected group, 61 mice were inoculated with 500 blood trypomastigotes via IP. The rate of mortality was over 50%, and the cause of death was sudden death due to cardiac failure. For the healthy control group, 24 mice were administered with a physiological saline solution via IP.

The animals were group-housed in an environmentally regulated vivarium, where they had free access to food and water. All animals were monitored daily to supervise their health. The animal procedures were supervised by a doctor of veterinary medicine trained in laboratory animal procedures and clinical evaluation. A veterinarian performed the anesthesia, diagnostic procedures, and humane endpoint, euthanized the animals, and performed all other laboratory animal procedures.

The humane endpoint was applied when the animal presented one or more of the following symptoms: i) weight loss greater than 20%, ii) dehydration greater than 10% (in case of bleeding wounds), iii) presence of unjustified pain and suffering, or iv) when the experiment came to an end, the necessary data had already been collected, and there was no justification for prolonging the experiment. The humane endpoint was administered by pentobarbital with a physiological saline solution via IP. For more detail on the murine model, see S2 Appendix.

### Multimodal data acquisition

For the murine model, 97 mice were employed. For the acute phase, 51 animals were studied (23 healthy control and 28 infected). For the chronic phase, 46 animals were analyzed (24 healthy control and 22 infected). Multimodal data were acquired from the acute group at 5, 15, 25, and 35 days post-infection for the acute phase. In contrast, for the chronic phase, acquisitions were done at 30, 60, 90, and 120 days. The data were acquired from four diagnostic techniques: ECG, ECHO, DOPPLER, and ELISA.

For ECG data acquisition, the mice’s cardiac activity was recorded by non-invasive ECG equipment (Mouse Specifics Inc., Quincy, MA, USA) for conscious animals using lead II. For each animal, 14 variables were recorded using EzCG Analysis Software package (Mouse Specifics Inc., Quincy, MA, USA). The extracted variables were PQ, QTc, PR, QT, RR, ST intervals, QRS complex, heart rate (HR), heart rate variability (HRV), QTc dispersion, percentage of cardiac variability (CV%), SR mean, and R amplitude mean.

For ECHO data acquisition, the mice were anesthetized using inhaled anesthesia (Patterson Scientific, Waukesha, WI, USA) at an induction dosage of 3% isoflurane and 0.5 L/min O2 using a chamber and maintained through a face mask with a dosage of 1.5–2.5% and 0.5 L/min O2. ECHO was performed using a 22 MHz lineal transducer and Mylab Seven equipment (ESAOTE S.P.A., Florence, Italy). Five variables were recorded: heart rate (HR), left ventricle diameter at the end systole (LVs), left ventricle diameter at the end diastole (LVd), fractional shortening (FS), and the ejection fraction (EF), which was calculated by the Teichholz equation [44].

Similarly, blood flow records using a Doppler system for mice (INDUS Doppler System) were recorded under anesthesia. A set of signals at the level of the mitral valve (MV) and ascending aorta (AO) were acquired by a 10 MHz transducer. In contrast, a set of signals at the level of the abdominal aorta (AbAO) were acquired using a 20 MHz transducer. For each mouse, 45 variables were captured related to heart rate, peak velocity, flow velocity, pulsation, and acceleration.

For ELISA, a set of duplicate antibody-detection tests were conducted. A blood sample was extracted from each animal by cardiac puncture performed under anesthesia. Each animal was anesthetized with a combination of ketamine and xylazine and then immediately euthanized by cervical dislocation. Afterward, three subclass antibody concentrations were recorded: IgG total (IgGT), IgG1, and IgG2a.

For each animal, 67 variables were recorded for the healthy control, acute infection, and chronic infection groups. The complete list of all extracted variables for each modality is presented in S3 Appendix.

### Imputation and augmentation of multimodal data from the proposed W–VAE

We propose an architecture based on VAE for imputing and augmenting data. VAE architecture is composing by the two neural networks: encoder (also known as *the inference model*) and decoder (also known as *the generative model*) [45]. The encoder is a neural network that takes an observed data point *x* ∈ *ℝ* and computes the posterior distribution $p_{\theta }(z|x)$ of the latent variables $z\in {\mathbb{R}}^{P}$, where *P* is the size of the latent space. Due to the unknown nature of $p_{\theta }(z|x)$, a simplified estimation of $q_{\phi }(z|x)$ is utilized [36].

The decoder is another neural network that takes the latent variables *z* to reconstruct the data points $\hat{x}$ by a generative model $p_{\theta }(x|z)$. Both networks use a Gaussian probability density in which the Gaussian mean and variance are parametrized by neural networks, with *ϕ* and *ϕ* containing the weights and biases of the neural networks of the encoder and decoder, respectively.

During the training, the neural network parameters *ϕ* and *ϕ* were optimized by the input data $X=(x_{1},x_{2},...,x_{n})$. This optimization occurs by minimizing the reconstruction loss (RL) and Kullback-Leibler (KL) divergence. The RL minimizes the mean square error between the reconstructed values $\hat{X}=(\hat{x_{1}},\hat{x_{2}},...,\hat{x_{n}})$ and the observed values $X=(x_{1},x_{2},...,x_{n})$. The KL minimizes the divergence between the variational posterior $q_{\phi }(Z|X)$, and the standard Gaussian prior *p*(*Z*). Both losses’ terms are represented in Eq (1):


lossVAE=∑n=1NEzn∼qϕ(zn|xn)[log ⁡ pθ(xn|zn)]⏟RL+KL(qϕ(zn|xn),p(zn)),⏟KLdivergence (1)

where the loss function of VAE is the sum of the RL term and the KL divergence term. This loss function is maximized by backpropagation through the hidden layers of the neural network, randomly sub-sampling the data at each training step and minimizing the loss through stochastic gradient descent [45].

We proposed a new loss function to improve the quality of generated data for VAE by implementing a masking mechanism and adding a weighting factor (WF). Based on this weighting, we called our proposed architecture W–VAE.

Firstly, the masking mechanism consists of generating synthetic imputations from the real multimodal data. The goal of this masking is to minimize the difference between real and the generated multimodal data. To do this, we replaced some real multimodal data with NaN values using different missing data rates: 10%, 20%, 30%, 40%, and 50%, as is common in the VAE literature [37,46].

**Fig 2:**
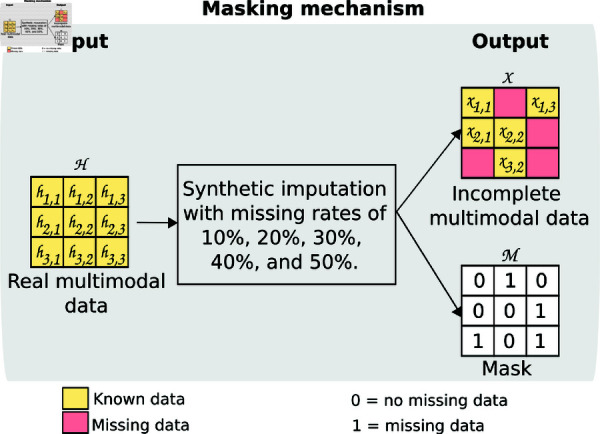
Workflow of the masking mechanism. Input is an *H* matrix of real multimodal data that is synthetically imputed with different missing rates. Output is an *X* matrix with missing data and an M binary mask matrix which indicates that, if *x_jk_* is known then *m_jk_*=0; otherwise, it is missing *m_jk_*=1.

The input of this mechanism is a matrix *H* with real multimodal data. Let *H* denote a matrix of real multimodal data (no missing data) with *j* individual *h*_1_,*h*_2_,...,*h_j_* and *k* features *F*_1_,*F*_2_,...,*F_k_*. Each feature corresponds to a variable in a specific modality (ECG, ECHO, DOPPLER, or ELISA). Using the *H* matrix, we selected a set of records randomly and replaced their original values with NaN. The number of records to replace depends on the missing rate used. The output are two matrices: i)an *X* matrix with missing data and ii) an *M* binary mask matrix. Let *X* denote a matrix with missing data with *j* individual *x*_1_,*x*_2_,...,*x_j_* and *k* features *F*_1_,*F*_2_,...,*F_k_*. We used *x_jk_* to indicate the value of the feature *k* in the individual *j*. The mask matrix *M*=*m_i_*,...,*m_n_* indicates which value in *X* is known; if *x_jk_* is known, then *m_jk_*=0, and otherwise it is missing *m_jk_*=1. Fig 2 illustrates the proposed masking mechanism process.

Secondly, to improve the quality of the generated data, we added a WF to the loss function (Eq 1). This factor consists of computing the accuracy from a classifier that uses the latent space *z* to predict the *Y * label. Let *Y * denote a matrix with the class labels from murine model with *j* individual *y*_1_,*y*_2_,...,*y_j_* and *c* classes. We used *y_jc_* to indicate the value of the class *c* in the individual *j*. In our proposal, *c* is equal to three classes: healthy individual, acute-phase individual, and chronic-phase individual. Specifically, we trained a classifier based on the Extremely Randomized Trees algorithm on the same decoder network [47]. The classifier was trained with 100 trees with a maximum depth of 4 and a maximum feature of base-2 logarithm; the quality of the split was measured with the *Gini* function. We used a grid search to select the optimal hyperparameters of this classifier.

We represent the weighted term as (1–*accuracy*) where the accuracy is obtained using the previously defined classifier. The goal of the term is to weight the network with i) a higher weight when the achieved accuracy by the classifier is low and ii) a lower weight when the accuracy is high. In this way, we weigh the network more when the generated data is of low quality, and we weigh it less when the quality is high. Finally, we add this weighted term to the loss function and rewrite as:


lossVAE=(∑n=1NEzn∼qϕ(zn|xn)[log ⁡ pθ(xn|zn)]⏟RL+KL(qϕ(zn|xn),p(zn))⏟KLdivergence)∗(1−accuracy).⏟WF (2)

We propose using a conditional VAE, which is an extension of a VAE described previously. To the conditional VAE we add a condition term, *cond*, which is both an encoder and decoder, to control the data generation so that we can generate controlled samples based on conditions. To generate these samples, the encoder is conditioned by two elements, *x* and *cond*, and is represented as $p_{\theta }(z|x,cond)$. Similarly, the decoder is conditioned by *z* and *cond* and is represented as $p_{\theta }(x|z,cond)$. To calculate the weights and biases, the condition term must also be added. In this proposal, the *cond* term is the class labels from the murine model (healthy individual, acute-phase individual, and chronic-phase individual).

**Fig 3:**
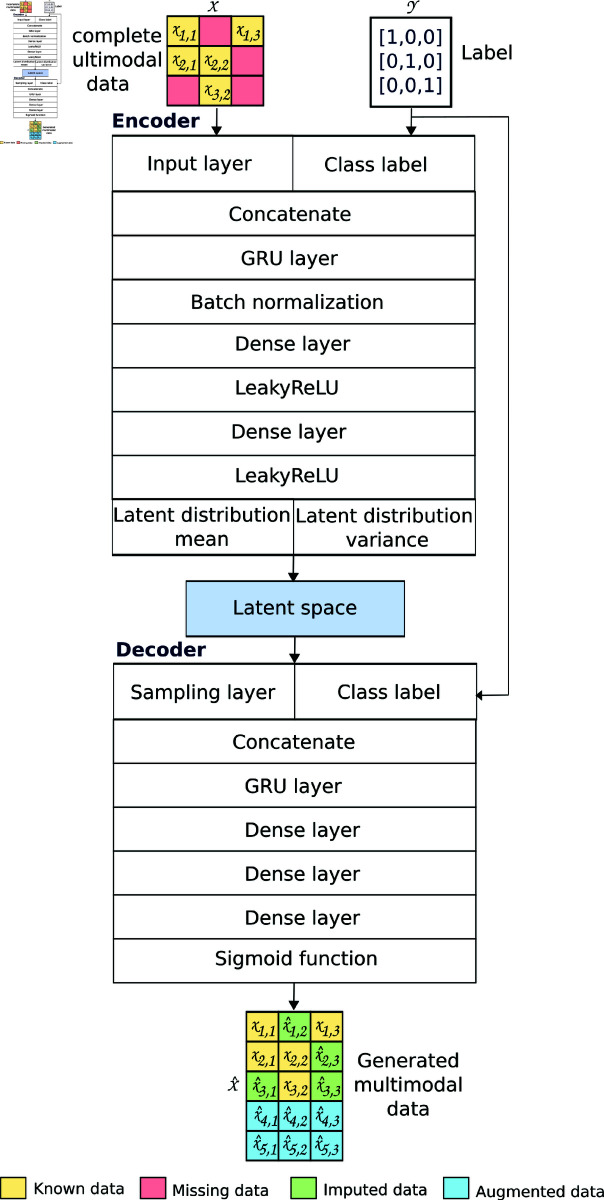
Overall architecture of the W–VAE proposed for imputing and augmenting data. W–VAE is conditioned by the labels from the murine model (healthy individual, acute-phase individual, and chronic-phase individual).

Fig 3 presents the general architecture of the proposed W–VAE. The inputs of the encoder network are the *X* matrix with missing multimodal data and the *Y * matrix with the labels. The matrices *X* and *Y * are concatenated and passed to a GRU layer with 128 features in the hidden state, four stacking layers, and a dropout layer of 0.3. We then added a batch normalization layer to prevent overfitting. Two dense layers with Leaky ReLU as an activation function in the intermediate layers were also added. The mean and standard deviation of the latent Gaussian distribution are computed by two dense layers. Both computations are reparametrized to generate the latent space.

The inputs of the decoder network were a reparametrized sample matrix and class labels. These inputs passed by a concatenate layer, two stacked GRU layers with 32 features in the hidden state, and three dense layers. A sigmoid function was used as an activation function, and the optimizer was Adam. We evaluated different hyperparameters of the encoder and decoder networks to identify the ideal scenario that would provide the model with the lowest loss function. From this, the batch size of the training set was 25, the dimension of latent space was 16, and the maximum epoch size was set to 1000 with an early stopping approach. The decoder output is a matrix $\hat{X}$ with data generated (imputed and augmented) from the network.

**Fig 4:**
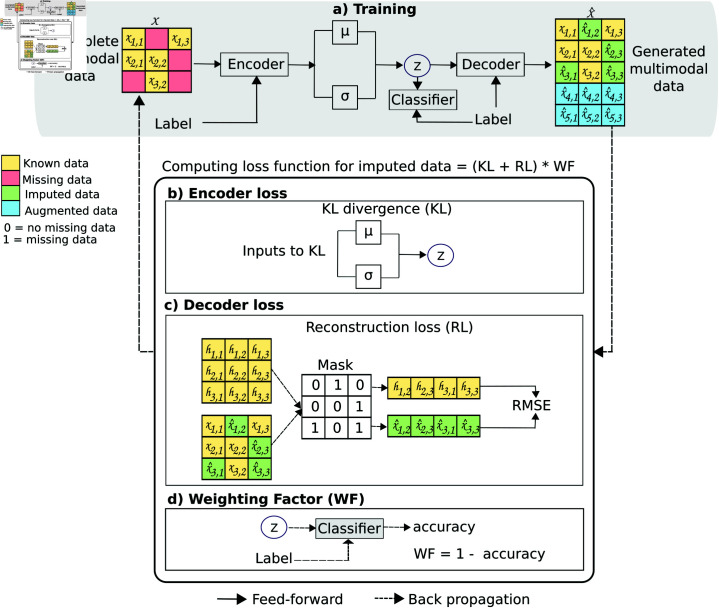
Workflow for training the proposed W–VAE.

Fig 4 shows the proposed workflow for training the conditional W–VAE. During feed-forward, the missing multimodal data *X* and class label *Y * were fed into the encoder network, and the network generated the latent space *z*. The decoder network takes *z* and *Y * to generate $\hat{X}$. During backward propagation, the encoder and decoder loss are computed, and the parameters *ϕ* and *ϕ* of the networks are adjusted to reduce the error. Both parameters are using to compute the KL divergence (Fig 4b).

We then computed the RL term of decoder loss using the M binary mask matrix obtained from the masking mechanism (Fig 4c). Using the mask *M*, we recovered the real multimodal data *w_jk_* and the generated multimodal data ${\hat{x}}_{jk}$ marked as missing (*m_jk_*=1). We then computed the root mean square error (RMSE) in Eq 3 from the recovered data:


$$\begin{array}{ccc} RMSE=\sqrt{\overset{n}{\underset{j=1}{\sum }}\frac{{\left(w_{jk}-{\hat{x}}_{jk}\right)}^{2}}{n},} & & \end{array}$$ (3)

where *w_jk_* are the real multimodal data, ${\hat{x}}_{jk}$ are the generated data, and *n* is the number of observations (Fig 4c). Finally, we computed the WF using the latent space *z* and label *Y *, which was added to the loss function (Fig 4d).

### Multi-classification of *T. cruzi* infection

We trained different multiclass classifiers for identifying healthy individuals, acute-phase individuals, and chronic-phase individuals with *T. cruzi* infection. First, we performed feature-wise normalization on each sample by subtracting the mean and dividing it by the standard deviation. Secondly, we explored the performance of classifiers on the real and generated multimodal data by the proposed W–VAE. We evaluated different feature-selection methods: i) sequential backward selection (SBS), ii) mutual information (MI), iii) principal component analysis (PCA), and iv) voting. We selected these methods because they are the most widely used on biomedical data [48,49].

SBS is a method that selects a subset of features based on removing one feature at a time from the cross-validation score of an estimator [50]. MI calculates the dependency of each feature with the target value, so the selected features are those that have the higher scores, which means more dependent variables [51]. PCA is a statistical method that transforms high-dimension data into lower dimensions while retaining as much information as possible through the computation of the principal components [52]. These components maximally explain the variance of all the variables. Finally, voting is a method that usually consists of selecting the most influential and relevant features obtained from other feature-selection methods to reduce the redundancy of the features [53–55]. In this proposal, our voting method consists of selecting the common features obtained between the SBS and MI methods. We train and evaluate the multimodal data using the following algorithms: Support Vector Machines (SVM), Random Forest (RF), Extremely Randomized Trees (ETC), Logistic Regression (LR), and Gaussian Process Classifier (GPC). We did hyperparameter tuning to select the optimal set of settings for each classifier. For more detail on the hyperparameter evaluated, see S4 Appendix.

### Evaluation of multiclass classifiers

**Fig 5:**
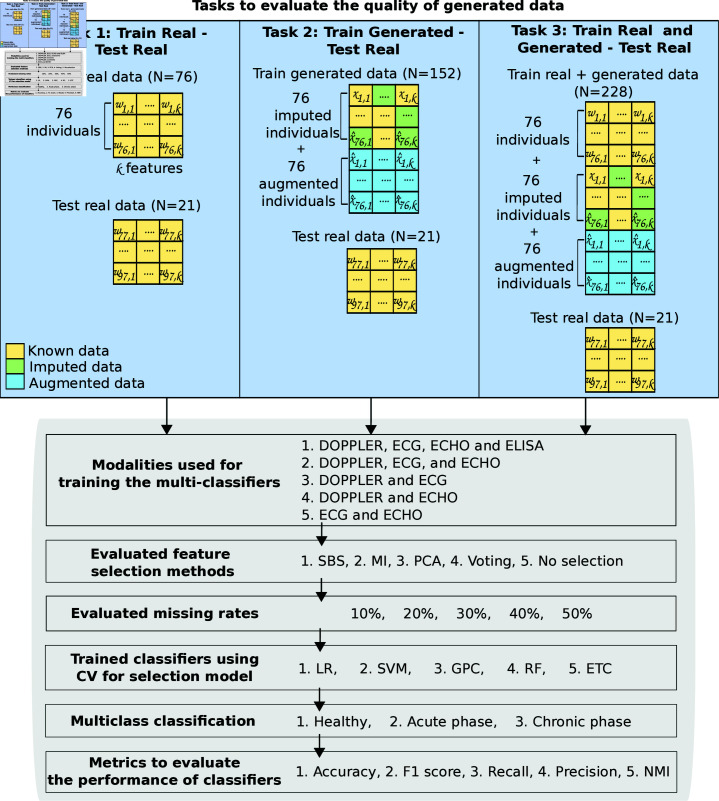
Workflow to evaluate the quality of the data generated from the proposed W–VAE in the multi-classification of healthy individuals, acute-phase individuals, and chronic-phase individuals with *T. cruzi* infection. ETC: Extremely Randomized Trees, GPC: Gaussian Process Classifier, MI: Mutual Information, PCA: Principal Component Analysis, RF: Random Forest, SBS: Sequential Backward Selection, SVM: Support Vector Machines.

We propose a workflow to evaluate the quality of generated data by W–VAE described in Fig 5. In this workflow we define three tasks for training different multiclass classifiers to identify healthy individuals and individuals in the acute or chronic phases of infection. First, we randomly split the *H* matrix with real multimodal data into non-overlapping training of real and testing real sets, consisting of 80% and 20%, respectively. Each task was configured as follows. In Task 1, we trained classifiers with the training real dataset and tested with the testing real dataset. In Task 2, we trained with the generated multimodal data by the proposed W–VAE and tested with the testing real dataset. Finally, in Task 3, we united the training real dataset with the generated multimodal data for training the classifiers and tested with the testing real dataset. All classifiers were evaluated on the same testing real dataset.

Each classifier was trained with different modalities, feature-selection methods, data modalities, and missing rates. To evaluate the effect of modalities on the multi-classification, we group the modalities as follows: 1) DOPPLER, ECG, ECHO, and ELISA, 2) DOPPLER, ECG, and ECHO, 3) DOPPLER and ECG, 4) DOPPLER and ECHO, and 5) ECG and ECHO. The trained classifiers were LR, SVM, GPC, RF, and ETC with the feature-selection methods of SBS, MI, PCA, and voting, as well as without feature selection. Each classifier was also trained with different missing rates of 10%, 20%, 30%, 40%, and 50%. To measure the performance, we computed the metrics of accuracy, F1 score, recall, precision, and normalized mutual information (NMI). This metrics are described in S5 Appendix. For classifier selection, we relied on grid search, comparing the prediction performance by five repetitions of stratified five-fold cross-validation.

## Results

### Task 1: Train real data – Test real data

**Fig 6:**
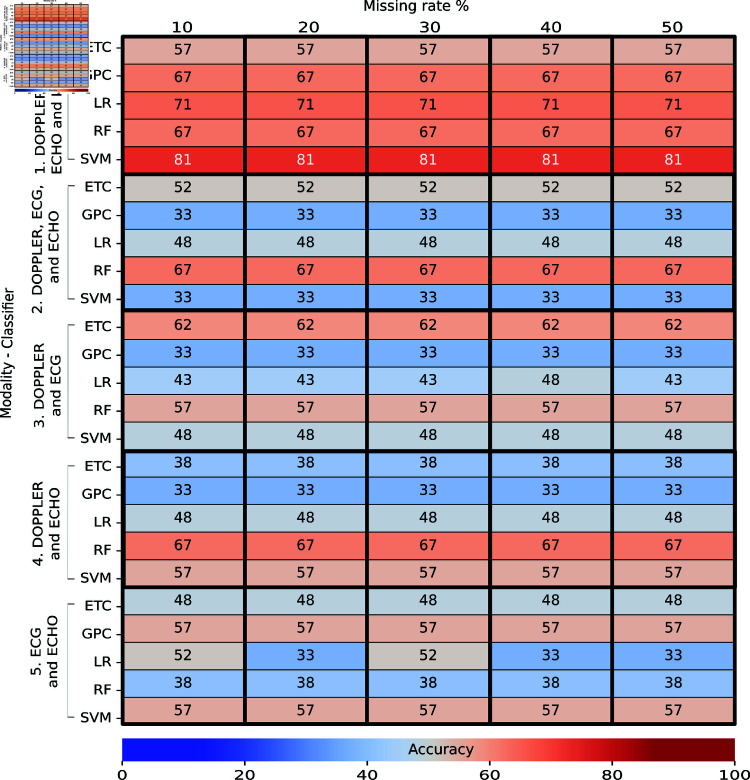
Performance of multi-classifiers in Task 1: Train real – Test real (without feature selection) as a percentage. Each column shows an evaluated missing rate, and each row indicates the modality used to train a specific classifier. The intersection shows the accuracy obtained during the final test. The color indicates whether the accuracy is high (closer to red) or low (closer to blue).

**Fig 7:**
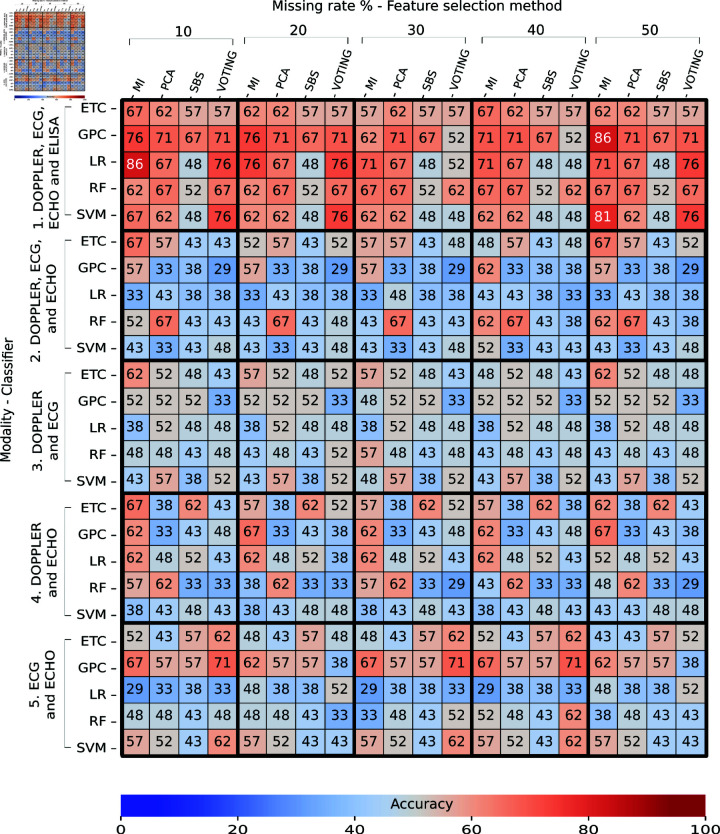
Performance of multi-classifiers in Task 1: Train real – Test real (with feature selection) expressed as a percentage. Each column shows a missing rate and a feature selector evaluated. Each row indicates the modality used to train a specific classifier. The intersection shows the accuracy obtained during the final test.

Figs 6 and 7 present the obtained accuracy of trained classifiers with real data and tested with real data in Task 1. A total of 76 individual were used to train the classifiers, and 21 individuals were used for testing. Fig 6 describes the performances of classifiers without feature selection, and Fig 7 shows the performances with feature selection. Each column corresponds to a missing rate and a feature-selection method evaluated (Fig 7). Each row indicates the modality used to train a specific classifier. The intersection between row and column shows the obtained accuracy as a percentage during the final test. The color indicates whether the accuracy is high (closer to red) or low (closer to blue). The multi-classification performance obtained in the cross-validation during the training, expressed as a mean and standard deviation, is shown in S6 Appendix.

At the top of both figures, we observe heatmaps with the presence of orange, which means accuracies above 60% using the modalities DOPPLER, ECG, ECHO, and ELISA. In the rest of the figures, we observed a greater presence of blue, which means accuracies of less than 60% with any combination of modalities, feature selections, or missing rates.

In the case of classifiers without feature selection (Fig 6), we detected that the SVM classifier trained with the DOPPLER, ECG, ECHO, and ELISA modalities achieved a high accuracy of 81% for all evaluated missing rates in the test set; this classifier, however, obtained a high variance of 72% ± 12% in training.

In contrast, a slightly better performance is observed in the LR classifier, which achieved an accuracy of 78*%* ± 11*%* in training and 71% in testing with the same modalities and missing rate as SVM. For classifiers with feature selection (Fig 7), the LR trained with the DOPPLER, ECG, ECHO, and ELISA modalities, MI as feature selector, and a missing rate of 10% achieved a high accuracy of 86% in testing and 84*%* ± 1*%* in training. We also detected that GPC trained with a missing rate of 50% in the same four modalities achieved an accuracy of 86% in testing, but this classifier obtained an accuracy of 86*%* ± 11*%* in training, which means that the classifier is sensitive to variations in the training data and may be overfitting. From both results, the accuracy of classifiers improved slightly with feature-selection methods in training and testing. We did notice, however, many classifiers with accuracies under 50% trained with modalities that did not include ELISA.

### Task 2: Train generated data – Test real data

**Fig 8:**
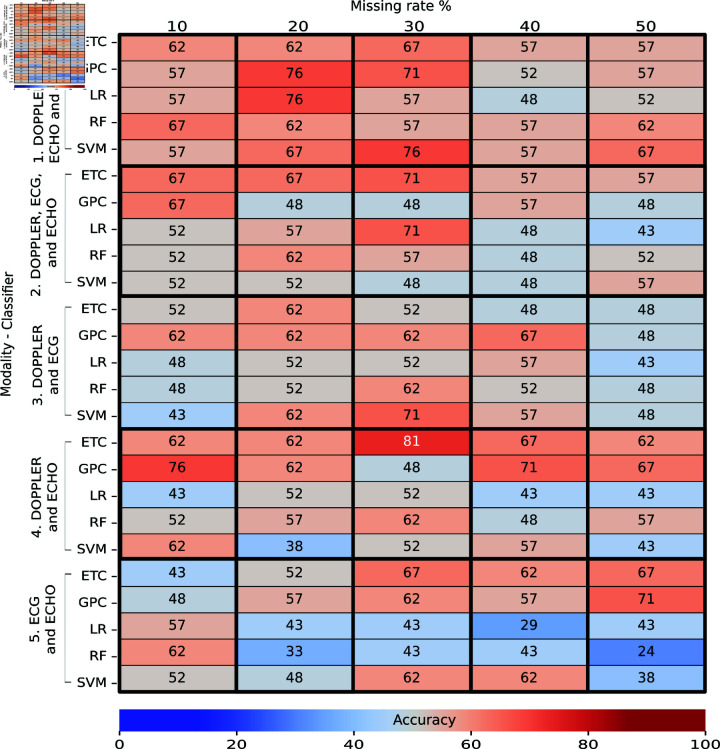
Performance of multi-classifiers in Task 2: Train generated – Test real (without feature selection) expressed as a percentage. The intersection between the rows and columns shows the accuracy obtained during the final test.

**Fig 9:**
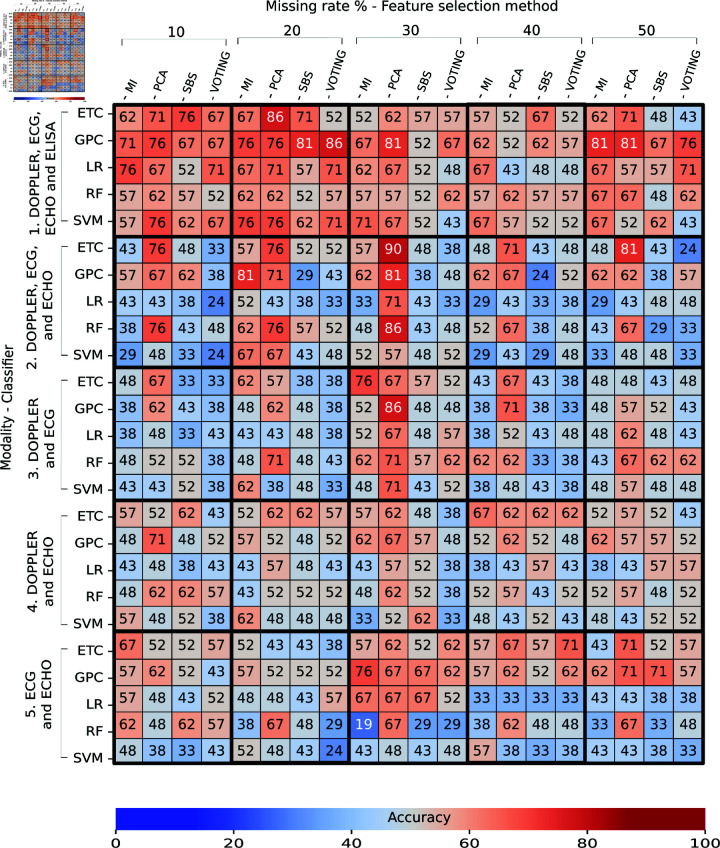
Performance of multi-classifiers in Task 2: Train generated – Test real (with feature selection) expressed as a percentage. The intersection between the rows and columns shows the accuracy obtained during the final test.

Figs 8 and 9 present the obtained accuracy of the trained classifiers with the generated data using W–VAE and tested with real data in Task 2. A total of 152 generated individual were used to train the classifiers, and 21 individuals were used for testing. The 152 generated individuals were obtained as follows: 76 imputed individual and 76 augmented individuals. In both figures we observe an increase of classifiers with accuracies of over 70%, which appear in light red on the heat maps. This is reflected in the decrease in blue in classifiers not trained with the ELISA modality compared with Task 1. We also observed accuracies of over 71% with missing rates of 10% with all modalities with and without feature selection. The multi-classification performance obtained in the cross-validation during the training, expressed as a mean and standard deviation, is shown in S7 Appendix.

For classifiers without feature selection (Fig 8), we observed that ETC trained with the DOPPLER and ECHO modalities and with a missing rate of 30% achieved an accuracy of 95%  ±  3% in training and 81% in testing. Interestingly, we observed high accuracies (over 99%) in training and accuracies of over 50 in testing, which means an overfitting problem where the classifier learned patterns very close to training set that cannot be applied to the test set.

For classifiers with feature selection (Fig 9), we observed that ETC trained with the DOPPLER, ECG, and ECHO modalities, PCA as the feature selector, and a missing rate of 30% achieved an accuracy of 82*%* ± 5*%* in training and 90% in testing. We also noticed a total of four classifiers with an accuracy of 86% with missing rates of 20% and 30%. From these, two classifiers (ETC and GPC) trained with DOPPLER, ECG, ECHO, and ELISA and a missing rate of 20% achieved an accuracy of 86*%* ± 4*%* and 88*%* ± 6*%* in training and testing, respectively. Another classifier (RF) trained with the DOPPLER, ECG, and ECHO modalities with a missing rate of 30% achieved an accuracy of 82*%* ± 6*%* in training; and the last classifier (GPC) was trained with DOPPLER and ECG modalities with a missing rate of 30% and achieved an accuracy of 79*%* ± 8*%* in training.

Overall, we observed an increase of classifiers with accuracies of over 80% with missing rates of 20% and 30%. In contrast, we detected low performance in classifiers trained with missing rate of 40% and 50% achieved accuracies under 80%. This means that classifiers trained with fewer missing data obtain better performance compared to classifiers trained with high missing rates. Additionally, we observed that voting method achieved the lowest accuracies with missing rates of 30%, 40%, and 50%.

Like in Task 1, we detected that training classifiers with feature selection achieved better performance than training classifiers without feature selection. In particular, we observed that classifiers without ELISA modality achieved accuracies of over 70% in all of Task 2. This mean that the modalities of DOPPLER, ECG, and ECHO contain features that contribute to detecting the phases of *T. cruzi* infection.

### Task 3: Train with real and generated data - Test real data

**Fig 10:**
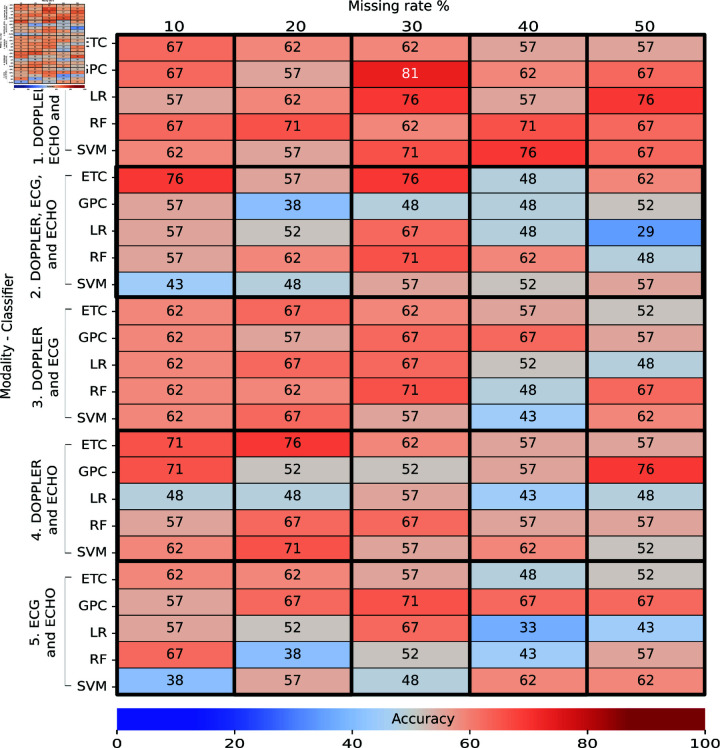
Performance of multi-classifiers in Task 3: Train with real and generated data – Test real (without feature selection) expressed as a percentage. The intersection between the rows and columns shows the accuracy obtained during the final test.

**Fig 11:**
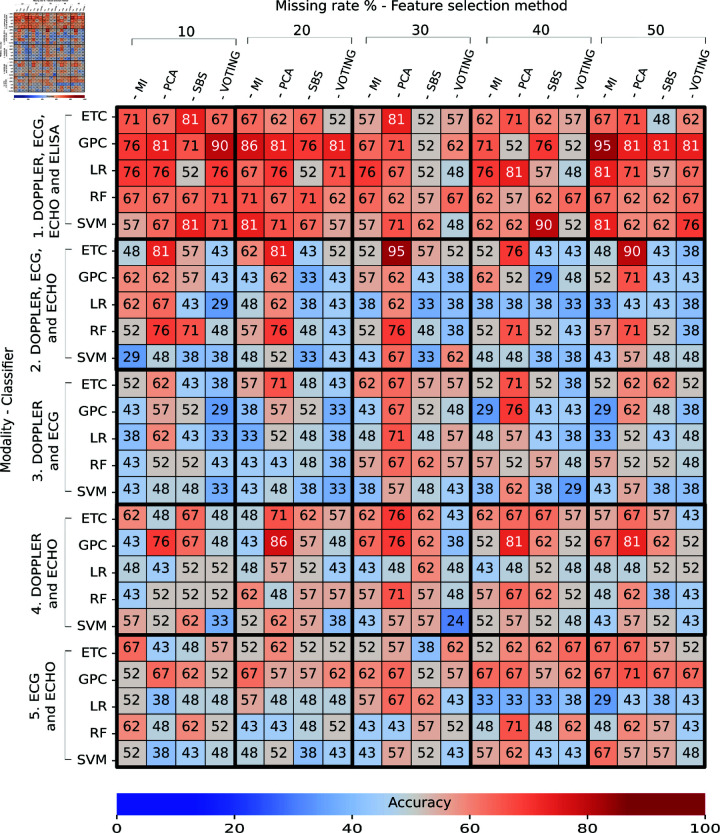
Performance of multi-classifiers in Task 3: Train with real and generated data – Test real (with feature selection) expressed as a percentage. The intersection between the rows and columns shows the accuracy obtained during the final test.

Figs 10 and 11 describe the obtained accuracy of all trained classifiers from the union of real data and generated data and tested with real data in Task 3. A total of 228 individuals were used to train the classifiers, and 21 individuals were used for testing. The 228 individuals were obtained as follows: 76 real individuals, 76 imputed individuals, and 76 augmented individuals. In both figures, we detected many classifiers with accuracies higher than 70% for all missing evaluated rates, which was reflected in the greater presence of orange and red on the heatmaps. For more information on the performance obtained in cross-validation during the training, see S8 Appendix.

For classifiers without feature selection (Fig 10), we found that the best accuracy was obtained by a GPC trained with a missing rate of 30% and with the DOPPLER, ECG, ECHO, and ELISA modalities achieved an accuracy of 93*%* ± 4*%* in training and 81% in testing. We identified high performances in all missing rates (with accuracies over 76%). Concerning modalities, we detected low performances in classifiers trained with ECG and ECHO (with accuracies around 60%). Notably, we detected fewer classifiers with accuracies under 50% in testing: these appear in blue. This means that increasing the amount of data (individual and generated) improved the performance of the classifiers by reducing overfitting.

For classifiers with feature selection (Fig 11), we identified that a GPC trained with MI as the feature selector, a missing rate of 50%, and the DOPPLER, ECG, ECHO, and ELISA modalities achieved an accuracy of 92*%* ± 4*%* in training and 95% in testing. Similarly, an ETC trained with PCA, a missing rate of 30%, and the DOPPLER, ECG, and ECHO modalities obtained an accuracy of 80*%* ± 4*%* in training and 95% in testing.

We observed a greater number of classifiers with accuracies of over 90% (red) and a smaller number of classifiers with accuracies of over 40% (blue). Concerning modalities, we observed that the lowest performances were obtained with classifiers trained with DOPPLER and ECG with any feature selection, classifiers, or missing rates (marked in blue). In contrast, the highest performances were obtained with classifiers trained with the DOPPLER, ECG, ECHO, and ELISA modalities (marked in red). We also detected good performances with the modalities: i) DOPPLER, ECG, and ECHO and ii) DOPPLER and ECHO (marked in orange). Regarding the feature selector, the highest performances were achieved with PCA and MI. We also observed that the voting method achieved the lowest performances (under 40%) with missing rates of 50%. Like in Task 1 and Task 2, the classifiers with feature-selection methods achieved better performances than those without feature selection. We also detected good performance with high missing rates.

### Classifier performance comparison and feature analysis

Table 1 presents the Top three classifiers with the best performance for each evaluated task. The first column describes the number of tasks, where 1 refers to ‘Task 1: Train Real – Test Real’, 2 refers to ‘Task 2: Train Generated – Test Real’, and 3 refers to ‘Task 3: Train Real and Generated – Test Real’. The second column indicates the modalities used to train the classifier. The third column shows the feature-selection (FS) method. The fourth column shows the missing rate. The fifth column describes the classifier used for training. The sixth column shows results of cross-validation expressed as the mean and standard deviation during the training. The seventh column indicates the overall obtained accuracy by the classifier used during the final test. The eighth column describes the normalized mutual information (NMI), where 0 means no mutual information and 1 means perfect correlation between the data used for testing and the data predicted by the classifier. Finally, we describe the metrics of accuracy, recall, precision, and F1 score for each evaluated class: healthy, acute, and chronic.

**Table 1:** Summary of the best performances to classify healthy individuals, and individuals in the acute and chronic phases of *T. cruzi* infection during the final test.

Task	Modality	FS	MR	CLF	Training Acc	Testing Acc	Testing NMI	Class	Acc	Rec	Pre	F1
1	DOPPLER,							Healthy	86%	100%	70%	82%
	ECG, ECHO,							Acute	86%	57%	100%	73%
	and ELISA	MI	10	LR	84% ± 1%	86%	76%	Chronic	100%	100%	100%	100%
	DOPPLER,							Healthy	86%	100%	70%	82%
	ECG, ECHO,							Acute	86%	57%	100%	73%
	and ELISA	MI	50	GPC	86% ± 11%	86%	76%	Chronic	100%	100%	100%	100%
	DOPPLER,							Healthy	81%	100%	64%	78%
	ECG, ECHO,							Acute	81%	43%	100%	60%
	and ELISA	MI	50	SVM	86% ± 12%	81%	73%	Chronic	100%	100%	100%	100%
2	DOPPLER,							Healthy	90%	86%	86%	86%
	ECG, and							Acute	90%	86%	86%	86%
	ECHO	PCA	30	ETC	82% ± 5%	90%	75%	Chronic	100%	100%	100%	100%
	DOPPLER,							Healthy	86%	100%	70%	82%
	ECG, ECHO,							Acute	90%	71%	100%	83%
	and ELISA	PCA	20	ETC	86% ± 4%	86%	67%	Chronic	95%	86%	100%	92%
	DOPPLER,							Healthy	76%	100%	58%	74%
	ECG, ECHO,							Acute	76%	29%	100%	44%
	and ELISA	Voting	20	GPC	88% ± 6%	86%	76%	Chronic	100%	100%	100%	100%
3	DOPPLER,							Healthy	**95%**	**100%**	88%	**93%**
	ECG, ECHO,							Acute	**95%**	**86%**	**100%**	**92%**
	and ELISA	MI	50	GPC	**92% ± 4%**	**95%**	**87%**	Chronic	**100%**	**100%**	**100%**	**100%**
	DOPPLER,							Healthy	**95%**	**100%**	88%	93%
	ECG, and							Acute	95%	**86%**	**100%**	92%
	ECHO	PCA	30	ETC	80% ± 4%	95%	87%	Chronic	**100%**	**100%**	**100%**	**100%**
	DOPPLER,							Healthy	**95%**	86%	**100%**	92%
	ECG, ECHO,							Acute	90%	**86%**	86%	86%
	and ELISA	Voting	10	GPC	91% ± 3%	90%	75%	Chronic	95%	**100%**	88%	93%

Acc: Accuracy; CLF: Classifier; FS: Feature Selection; F1: F1 score; NMI: Normalized Mutual Information; MR: Missing Rate; Pre: Precision; Rec: Recall. Performance during the training set is shown with mean and standard deviation. Bold font indicates best result.

For Task 1, LR achieved the best performance, with an overall accuracy of 86% and an NMI of 76 % during the final test. This classifier was trained with the DOPPLER, ECG, ECHO, and ELISA modalities, MI as the feature selector, and a missing rate of 10%. For the healthy class, this classifier obtained an accuracy of 86%, recall of 100%, precision of 70%, and F1 score of 82%. For the acute class, the classifier achieved an accuracy of 86%, recall of 57%, precision of 100%, and F1 score of 73%. Finally, for the chronic class, the classifier achieved an accuracy of 100% for all the evaluated metrics. For this classifier, the best performance was achieved in the chronic phase, followed by the healthy and acute phases. We detected that the GPC and SVM classifiers trained with high missing rates (50%) present high variance compared with LR trained with a low missing rate (10%) during training.

For instance, GPC achieved an accuracy of 86*%* ± 11*%* and SVM obtained an 86*%* ± 12*%*, whereas LR achieved an accuracy of 84*%* ± 1*%*, which means less variance than GPC or SVM. On the other hand, we observed that the three classifiers with high accuracy in the test set were trained with the DOPPLER, ECG, ECHO, and ELISA modalities and MI as the feature selector. Additionally, we detected that all classifiers achieved a precision of 100% for chronic class. We also found that three classifiers achieved an NMI of over 73%, which means a high correlation between the real and test data.

For Task 2, the ETC classifier trained with PCA as the feature selector, a missing rate of 30%, and the four modalities achieved a high accuracy of 90% and an NMI of 75% in the test set. In this classifier, the best performance was achieved in chronic class (100% for all metrics), followed by the healthy and acute classes, with 90% accuracy. We observed that three classifiers in Task 2 achieved a low variance compared with Task 1 during training. For instance, the ETC classifier trained with a 30% missing rate obtained an accuracy of 82*%* ± 5*%*, whereas ETC classifier obtained an accuracy of 86*%* ± 4*%* when trained with a 20% missing rate, and GPC achieved an accuracy of 88*%* ± 6*%*. Of the three classifiers with the best performance in Task 2, two were trained with ETC and with PCA as the feature selector, and the third classifier was trained with GPC and voting. Concerning performance in classes, we detected that the GPC classifier achieved the lowest scores in the acute class (recall = 29% and F1 score = 44%) compared with ETC, which achieved high performance (recall and an F1 score over 70% in both metrics). Interesting, classifiers trained with high absence rates (30%) achieved better performance compared with classifiers trained with 20% of missing rate.

For Task 3, we observed that the classifier that achieved the highest performances from all tasks evaluated was a GPC trained with the DOPPLER, ECG, ECHO, and ELISA modalities, with a missing rate of 50% and MI as the feature selector. The accuracy obtained from this classifier during training was 92*%* ± 4*%* and 95% in the test set. This classifier achieved also the best performance in all evaluated metrics for each class. In the acute class, it achieved the highest accuracy (95%), recall (86%), precision (100%), and F1 score (92%). These results represented the highest performance in the acute phase compared to Task 1 and Task 2, which means that increasing the training set (with real and generated data) contributed to a better generalization of the classifier. For the chronic phase, the classifier achieved 100% for all evaluated metrics. Interestingly, this performance in the acute and chronic classes achieved by the GPC classifier was similar to that obtained by the ETC classifier trained with the DOPPLER, ECG, and ECHO modalities, with PCA as the feature selector and a missing rate of 30%.

On the one hand, we observed that three classifiers in Task 1 (LR, GPC and SVM) achieved 100% accuracy for the chronic class; this performance was equal to that obtained by GPC and ETC in Task 3. These results strengthen our intuition that the generated data share a similar distribution with real data and contribute to classifier performance. On the other hand, we noticed that the variance achieved was greatest in Task 1 and decreased in Task 2 and Task 3 during the training. These performances achieved mean that augmented data contributed to improving the performance and generalization of the classifiers. In addition, the distribution of the missing rates and trained classifiers was as follows: three classifiers were trained with missing rates of 50%, two classifiers were trained with a 30% missing rate, two classifiers were trained with a 20% missing rate, and two classifiers were trained with a 10% missing rate (see Table 1).

From all the evaluated classifiers, we found that the classifiers trained with feature selection achieved a better performance than those without. This performance is because, by reducing the dimensionality of the data, we obtained the most useful features for training a classifier and therefore improved its performance. The highest overall accuracy found was 95% using MI as a feature selector (Task 3) against the better performance of 81% without feature selection also in Task 3 (see Fig 10). Moreover, we found that the DOPPLER, ECG, ECHO, and ELISA modalities achieved the best performance both with and without feature selection. This was an expected result because the ELISA features indicate the antibodies present that are created against the infection.

Other interesting associated modality groups were: i) DOPPLER, ECG, and ECHO and ii) DOPPLER and ECHO. We observed that the trained classifiers with these groups achieved accuracies of over 70% (Task 2) and over 80% (Task 3). This means that these modalities contain features that can help identify the phases of *T. cruzi* infection.

**Fig 12:**
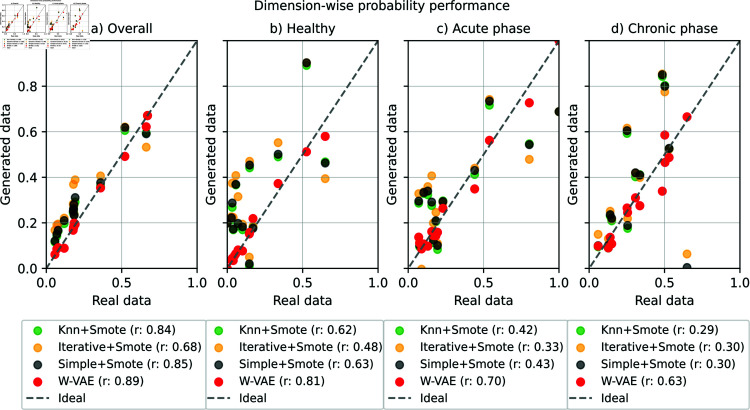
Comparison of dimension-wise probability and correlation coefficients between W–VAE and the state-of-the-art to impute and augment data. The x-axis is the Bernoulli success probability for the features of the real data sets, while the y-axis is the corresponding value from the generated data for each evaluated technique. Each blue dot represents a feature of the data set. The diagonal line indicates an identical Bernoulli success probability of both the real and generated data sets.

Overall, we found that better performance was achieved in the trained classifiers in Task 2 (imputed data) and Task 3 (augmented data) than in Task 1 (real data). This result indicates that: i) the imputed data are highly correlated with to real data and ii) increasing the training set with augmented data contributes to the generalization of the classifier. To validate this, we performed a dimension-wise probability comparison between the generated data and real data on test set (Fig 12). On this task, we used the GPC trained with MI and a missing rate of 50%, which achieved one of the highest performances on Task 3.

We also compared our W–VAE approach with state-of-the-art techniques to impute and augment data. To impute the data, we used three imputation techniques: i) KNN, ii) MICE, and iii) simple (where missing values were replaced by the mean). To augment the data, we used the SMOTE technique. We implemented these techniques, which are available in the Scikit Learn library in Python. For the MICE method, we used the implementation from Scikit Learn called *‘Iterative Imputer’* [56].

Fig 12 shows the results of comparing the state-of-the-art to impute and augment data with the proposed W–VAE architecture. Each plot shows the performance overall, for healthy individuals, and for the acute and chronic phases. In all these plots, we observed a consistency of features in the real data and the data generated by W–VAE because both sets are closer to the ideal probability (red dots).

On the overall plot, the highest correlation was for W–VAE (0.89), and the lowest was for iterative imputation with SMOTE to the augmented data (0.68). For healthy individuals, W–VAE achieved a high correlation (0.81) compared to the rest of the imputers (from 0.48 to 0.63). For the acute phase, a correlation of 0.70 was achieved by W–VAE, and a correlation of over 0.4 was achieved by the rest of the imputers. Finally, for the chronic phase, the W–VAE obtained 0.63—more than half of what the other techniques achieved. Specifically for this phase, we observed a slight difference compared to healthy and acute phases (Fig 12d). In particular, we found that some features can be far from the ideal. Considering that this classifier was trained with a 50% missing rate, the quality of the generated data obtained is acceptable. From all these results, we observed that the data generated by W–VAE shows a fidelity to the expectations and also achieved high correlations compared with the state-of-the-art. These results may indicate that the generated data show high quality compared with the real data. We also analyzed the probability density function of the real and generated data, which are described in S9 Appendix.

On the other hand, we studied the features selected as relevant, as identified by the MI method and used to train the GPC in Task 3. Table 2 describes the features selected, where each column describe the relevant features for each modality.

**Table 2:** Features selected computationally as relevant in Task 3. The trained classifier with these features achieved among the highest performances of all experiments done on the test set.

DOPPLER	ECG	ECHO	ELISA
- AbAO Resistive Index Avg - AO Stroke Distance SD - AbAO HR SD - AO HR SD - AO Ejection Time SD - AbAO RR interval SD
- CV% - QTc dispersion - HRV
- LVs
- lgG2a - lgGT - lgG1

AO: Aorta; AbAO: Abdominal Aorta; Avg: average; HR: heart rate; HRV: heart rate variability; IgG: Immunoglobulin G. LVs: Left ventricle systole; QT interval: ventricular depolarization and repolarization.

Overall, we observed that the AbAO Resistive Index Avg feature for the DOPPLER modality was selected as important, and it had already been identified as relevant to infection with *T. cruzi* [10]. Resistive index is an indirect measurement (peak systolic velocity-end diastolic velocity) / peak of systolic velocity) of the resistance of the arterial flow. Experimental studies in mice have demonstrated that the parasite invades the endothelial cells from the aorta, causing vasculitis and resulting in dysfunction in the contractility and relaxation of the mouse aorta and ultimately a vascular dysfunction [57]. The parameters of cardiac variability (CV%) and heart rate variability (HRV) are correlated, and they reflect the capacity for variation of the heart rate, which is controlled by the autonomic nervous system. The alteration in those variables suggests autonomic dysfunction. HRV changes in humans in the chronic stage of the disease has been associated with a risk of sudden death, similar to alterations in the QTc dispersion variable [58].

On the other hand, LVs (Left ventricular end systolic volume) alteration can be the result of concentric hypertrophic changes due systemic hypertension, or stiffness of the myocardium because of cardiac muscular fibrosis or dilatation during end-stage cardiac disease. The presence of antibodies during the infection (IgG2a, IgGT and IgG1) is the immune system response against the parasite. Antibodies against the parasite appear during the first week of infection and rise as the disease evolves.

Overall, we observed that the features selected computationally as relevant are associated with clinical findings in the acute and chronic phases of *T. cruzi* infection. Therefore, we argue that the W–VAE architecture proposed can generate data that are similar in quality and complexity to the real data and could be replicated in other domains.

## Discussion

This work presented a new strategy for classifying healthy individuals and individuals in the acute and chronic phase of infection by *T. cruzi* with missing and limited data applied to a murine model. To attend these issues, we proposed a weighted deep generative approach based on a Variational Auto-Encoder, called W–VAE, to impute and augment data.

We implemented a loss function and masking mechanism to improve the quality of the data generated by the proposed W–VAE. We have demonstrated that the proposed architecture outperforms the current state-of-the-art imputation and augmentation data techniques. In particular, we have demonstrated that the data generated by the W–VAE achieved the highest positive correlation, in all phases of infection, compared with the most common imputation techniques, such KNN, iterative, and simple imputation. We also observed that the distribution of the generated data by W–VAE was adjusted to the optimal compared to the data generated by SMOTE.

The data generated by the W–VAE were evaluated on three different tasks to compare their performance and robustness in the multi-classification of the phases of *T. cruzi* infection. In extensive experiments with different modalities, missing rates, and feature-selection methods, we trained several classifiers with real and generated data. We observed that the classifiers trained with imputed and generated data demonstrated a significantly superior performance compared with classifiers trained only with real data. Moreover, we detected that these high performances existed not only at a general level but also in each of the evaluated classes (healthy, acute, and chronic). These results strengthen our intuition that the generated data share a similar distribution with real data and contribute to classifier performance.

We provided evidence of the effect of the missing rate on multiclass classification performance. We found that classifiers trained with high missing rates (over 30%) achieved high, even superior, performance(>90%) compared with classifiers trained with low missing rates. Previous works have shown that architectures based on VAE for imputation data obtained high performances with high missing rates (>30%). For instance, [38] presents a VAE to impute carotid artery lesion data; in that work, the authors achieved 85% accuracy with a missing rate of 30% for the early identification of abnormal arteries compared with imputations using a mixture of mean and KNN. Similarly, [37] achieved the lowest RMSE(<0.3) in the task of imputing RNA sequencing data compared with KNN with missing rates of 30% using an extension of a VAE. Finally, [39] reported an accuracy of 97% with a missing rate of 80% (the best performance) and showed accuracies of 88% with missing rates of 40% and 60% using a proposed VAE for imputing medical data.

Deep learning architectures, such as the proposed W–VAE, are capable of capturing complex high-dimensional data structures because these architectures comprise multiple stacked layers, where each layer is responsible for discovering hierarchical patterns in the data that is used in each of the following layers. Each layer is responsible for a task, then each layer is optimized to accomplish its task during training in such a way that the entire deep model can discover sophisticated characteristics and relationships. Fig 3 presents the general architecture of the proposed W–VAE, which is composed of two networks: *encoder* and *decoder*, where each network composes a set of stacked layers. The *encoder* network takes an observed data set as input and transforms it into a latent space. The *decoder* takes this latent space to reconstruct the data set. Using this architecture in combination with the loss function and masking mechanism proposed, we achieved high performance (accuracy >95%) in the classification of individuals: healthy, acute, and chronic.

By comparing our performance achieved with the state-of-the-art, we found that the obtained performance is competitive with that reported in the state-of-the-art in the multi-classification of healthy individuals, acute-phase individuals, and chronic-phase individuals with *T. cruzi*. In the multi-classification using only ECG modality and without missing data, Haro et al. [11] achieved an accuracy of 91.3%, whereas Valdez et al. [12] reported an accuracy of 98%. In contrast to these works, we presented the results of a W–VAE approach to impute and augment data, achieving an overall accuracy of 95% in the multi-classification from four diagnostic techniques: ECG, ECHO, DOPPLER, and ELISA. As can be seen, few works have addressed the problem of missing data to classify healthy individuals and the different phases of *T. cruzi* infection in multimodal data. To our knowledge, this is the first work that explores data augmentation to study *T. cruzi* infection.

Our study has some limitations. We studied the two clinical phases of *T. cruzi* infection in a murine model. In previous studies, the murine model has been proven useful for the study of *T. cruzi* infection, to test drugs for its treatment, and for the development of therapeutic vaccines against it [59,60]. The advantage of this murine model is that it allows the study of the acute and chronic phases of the disease in a short period of time. On the other hand, the number of animals employed was limited due the difficulties of working with biological models and their response to an infectious agent. We also performed only a basic evaluation of systolic function due to equipment limitations. We did, however, include the ejection fraction, fractional shortening, and the dimensions of the left ventricle in diastole and systole (see S3 Appendix to more information about the extracted features). We also had a limited number of training and testing samples.

In future work, we intend to incorporate other multimodal data, such as images processing from ECHO, as well as to evaluate other deep learning architectures for multi-classification. In addition, we intend to use the proposed W–VAE as feature selector. In this work, however, we have focused on novel contributions in the deep-learning field to deal with missing and scarce multimodal data in particular. As part of this work, we analyzed the features selected as relevant to the classifier with the best performance. The objective of this analysis was to better understand the pathophysiology of infection and study the progression of the organ damage caused by *T. cruzi*.

## Conclusion

In this work, we presented a new W–VAE approach to impute and augment multimodal data. We implemented a new loss function by adding a weighing factor and a masking mechanism to improve the quality of the data generated by the proposed W–VAE. To evaluate the quality of the imputed and generated data from the W–VAE, we performed a multi-classification to identify healthy individuals and individuals in the acute and chronic phases of *T. cruzi* infection in a murine model. This work arises in the context of missing and limited data: common issues with multimodal data in the biomedical domain. Having access to large databases with completed multimodal data is a particular challenge due to the lack of awareness and understanding of *T. cruzi* infection. The obtained results demonstrated that the proposed W–VAE architecture can be useful in data generation, especially in studying the phases of *T. cruzi* infection. We then consider that our proposed architecture can be replicated in other domains for imputed and augmented multimodal data. Our work contributes to the field by providing a tool to generate data and a methodology to identify possible heart damage from missing or limited multimodal data.

## Supporting information

S1 Appendix Acronyms list. (PDF)

S2 Appendix Murine model description. (PDF)

S3 Appendix Features extracted for each modality from the murine model (PDF)

S4 Appendix Hyperparameters tuning for multi-classification. (PDF)

S5 Appendix Metrics used to evaluate the performance of multi-classification. (PDF)

S6 Appendix Performance of multi-classifiers during training in Task 1. (PDF)

S7 Appendix Performance of multi-classifiers during training in Task 2. (PDF)

S8 Appendix Performance of multi-classifiers during training in Task 3. (PDF)

S9 Appendix Analysis of probability density function. (PDF)
