# Supplementary material for: Weighted–VAE: A deep learning approach for multimodal data generation applied to experimental T. cruzi infection
Source: PLoS One. 2025 Mar 24;20(3):e0315843. doi: 10.1371/journal.pone.0315843 (PMC11932709; doi:10.1371/journal.pone.0315843)
Supplement: S1 Appendix — (PDF) [file pone.0315843.s001.pdf]

Weighted–VAE: A Deep Learning Approach for Multimodal Data Generation Applied to Experimental *T. cruzi* infection

Blanca Vazquez*, Nidiyare Hevia-Montiel, Jorge Perez-Gonzalez, Paulina Haro.
* Corresponding author: blanca.vazquez@iimas.unam.mx

S1 Appendix: Acronyms list

- AbAO: Abdominal Aorta
- Acc: Accuracy
- AO: Ascending Aorta
- Avg: average
- CD: Chagas Disease
- CLF: Classifier
- CV%: Percentage of Cardiac Variability
- ECG: Electrocardiography
- ECHO: Echocardiography
- EF: Ejection Fraction
- ELISA: Enzyme Linked Immunosorbent Assay
- ETC: Extremely Randomized Trees
- DOPPLER: Spectral Ultrasound Doppler
- FS: Feature Selection
- GPC: Gaussian Process Classifier
- HR: Heart Rate
- HRV: heart Rate Variability
- IgG: Immunoglobulin G
- IgG1: Immunoglobulin IgG1
- IgG2a: Immunoglobulin IgG2a
- IgGT: Immunoglobulin total
- IP: Intraperitoneal
- KL: Kullback-Leibler divergence

- LR: Logistic Regression
- LVd: Left Ventricle diameter at the end diastole
- LVs: Left Ventricle diameter at the end systole
- PCA: Principal Component Analysis
- QT interval: ventricular depolarization and repolarization
- MI: Mutual Information
- MR: Missing Rate
- MV: Mitral Valve
- NMI: Normalized Mutual Information
- RF: Random Forest
- RL: Reconstruction Loss
- RMSE: Root Mean Square Error
- SBS: Sequential Backward Selection
- SD: Standard Deviation
- SVM: Support Vector Machines
- *T. cruzi*: *Trypanosoma cruzi*
- WF: Weighting Factor
- WHO: World Health Organization
- W–VAE: It is a weighted deep generative architecture based on a Variational Auto-Encoder.
