# Supplementary material for: Weighted–VAE: A deep learning approach for multimodal data generation applied to experimental T. cruzi infection
Source: PLoS One. 2025 Mar 24;20(3):e0315843. doi: 10.1371/journal.pone.0315843 (PMC11932709; doi:10.1371/journal.pone.0315843)
Supplement: S2 Appendix — (PDF) [file pone.0315843.s002.pdf]

Weighted–VAE: A Deep Learning Approach for Multimodal Data Generation Applied to Experimental *T. cruzi* infection

Blanca Vazquez*, Nidiyare Hevia-Montiel, Jorge Perez-Gonzalez, Paulina Haro.
* Corresponding author: blanca.vazquez@iimas.unam.mx

S2 Appendix: Murine model description

A murine model was realized to study the phases of *T. cruzi* infection. For the experimental acute phase infection, a total of 108 healthy female ICR mice were employed. At the beginning of the experiment, the mice were between 6 and 8 weeks old. In this phase, the experiment lasted 35 days. For the infected group, 66 mice were inoculated with 1000 blood trypomastigotes of strain H1 (TcI lineage) *T. cruzi* via intraperitoneal (IP). In the infected group, a total of 16 mice died which represented a mortality rate of 24%. The causes of death and the number of deceased animals were: i) 6 mice dead for humanitarian endpoint due respiratory distress, dehydration, and hypothermia (poor perfusion), and ii) 10 mice were found dead due cardiac failure and sudden death (before meeting criteria for euthanasia). For the healthy-control group, 42 mice were administered with a physiological saline solution IP. Multimodal data were acquired on 6 infected and 6 healthy-control animals per sampling period.

For the experimental chronic phase infection, a total of 85 healthy female ICR mice were employed. At the beginning of the experiment, the mice were between 6 and 8 weeks old. In this phase, the experiment lasted 120 days. For infected group, 61 mice inoculated with 500 blood trypomastigotes via IP. The rate of mortality was over 50% in the infected group. Sudden death due cardiac failure occurred on 31 animals (before meeting criteria for euthanasia). For the healthy-control group, 24 mice were administered with a physiological saline solution via IP. Multimodal data were acquired on 6 infected and 6 healthy-control animals per sampling period.

The animals were group housed in polysulfonate cages (maximum 6 animals per cage) in an environmentally regulated vivarium. The cages were controlled for temperature (20-26°C), lighting (12 h light-dark cycle), and air exchanges with a capacity of 120 cubic feet per minute. The cages were attached to an automated isolation system with a system free of vibrations and noise. The dimensions of cages were 39 cm long, 20 cm wide, and 24 cm high, and these had shavings as bedding which was changed twice a week. The animals had free access to food and water. Related to food, mice were fed with Harlan brand pellets ad libitum. As entertainment, the cages had cardboard tubes, and the mice were kept in groups to promote socialization activities. All animals were monitored daily to supervise their health. In particular, the behavior, coat, water and food consumption were monitored throughout the experiment.

All animals used in the experiment were handled according to the Care and Use of Laboratory Animals Guide (eighth edition). The protocol was approved by the Ethics Committee of the Centro de Investigaciones Regionales Dr. Hideyo Noguchi (CIRB-006-2017) at the Universidad Autónoma de Yucatán, México. The animal procedures were supervised by a Doctor in veterinary Medicine, trained in laboratory animals procedures, and clinical evaluation. A veterinarian performed anesthesia, diagnostic procedures, humane endpoint, euthanized the animals, and performed all procedures on laboratory animals.

The humane endpoint was applied when the animal presents one or more of the following cases: i) weight loss greater than 20%, ii) dehydration greater than 10% (in case of bleeding wounds), iii) presence of unjustified pain and suffering, and iv) when the experiment comes to an end and the necessary data has already been collected and there is no justification to prolong the experiment. For the humane endpoint was administered Pentobarbital (210mg/kg) with a physiological saline solution 1:1 via IP. When the animals comes to an end these were anesthetized with a combination of Ketamine and Xylazine (10-13 mg/kg) and then immediately euthanized by cervical dislocation.
