# Supplementary material for: Weighted–VAE: A deep learning approach for multimodal data generation applied to experimental T. cruzi infection
Source: PLoS One. 2025 Mar 24;20(3):e0315843. doi: 10.1371/journal.pone.0315843 (PMC11932709; doi:10.1371/journal.pone.0315843)
Supplement: S3 Appendix — (PDF) [file pone.0315843.s003.pdf]

Weighted–VAE: A Deep Learning Approach for Multimodal Data Generation Applied to Experimental *T. cruzi* infection

Blanca Vazquez*, Nidiyare Hevia-Montiel, Jorge Perez-Gonzalez, Paulina Haro.
* Corresponding author: blanca.vazquez@iimas.unam.mx

S3 Appendix: Features extracted for each modality from the murine model.

We listed all extracted variables for each modality from the murine model in Table 1.

Table 1. Features to study the *T. cruzi* infection in a murine model

Modality	Feature		
ECG	1. HR<br>2. HRV<br>3. CV<br>4. RR interval<br>5. PQ interval	6. R interval<br>7. QRS<br>8. QT interval<br>9. ST interval<br>10. QTc interval	11. QTc<br>12. QTc dispersion<br>13. SR mean<br>14. R amplitude mean
ECHO	1. HR short axis<br>2. LVd	3. LVs<br>4. Ejection Fraction	5. Fractional Shortening
DOPPLER	1. AbAO HR Avg<br>2. AbAO HR SD<br>3. AbAO RR internal Avg<br>4. AbAO RR interval SD<br>5. AbAO Peak velocity Avg<br>6. AbAO Peak velocity SD<br>7. AbAO Minimum Flow Velocity Avg<br>8. AbAO Minimum Velocity SD<br>9. AbAO Mean Flow velocity Avg<br>10. AbAO Mean Flow velocity SD<br>11. AbAO Pulsability Index Avg<br>12. AbAO Pulsability Index SD<br>13. AbAO Resistivity Index Avg<br>14. AbAO Resistivity Index SD<br>15. AO HR Avg<br>16. AO HR SD	17. AO RR Interval Avg<br>18. AO RR Interval SD<br>19. AO Pre-ejection time Avg<br>20. AO Pre-ejection time SD<br>21. AO Peak velocity Avg<br>22. AO Peak velocity SD<br>23. AO Stroke Distance Avg<br>24. AO Stroke Distance SD<br>25. AO Ejection time Avg<br>26. AO Ejection Time SD<br>27. AO Rise Time Avg<br>28. AO Rise Time SD<br>29. AO Mean velocity Avg<br>30. AO Mean velocity SD<br>31. AO Mean Acceleration Time Avg<br>32. AO Mean Acceleration Time SD	33. AO Peak Acceleration Avg<br>34. AO Peak Acceleration SD<br>35. MV HR Avg<br>36. MV HR SD<br>37. MV RR Interval Avg<br>38. MV RR Interval SD<br>39. MV E Peak velocity Avg<br>40. MV E Peak velocity SD<br>41. MV E Acceleration time Avg<br>42. MV E Acceleration time SD<br>43. MV E Peak to Peak time SD<br>44. MV E Deceleration time SD<br>45. MV E Deceleration Rate SD
ELISA	1. IgGT	2. IgG1	3. IgG2a

For ECHO modality, the fractional shortening (FS) and the ejection fraction (EF) were calculating using the Teichholz Equation:

$$Vol = 7D^3/(2.4 + D) \tag{1}$$

where Vol is the Left Ventricle (LV) volume and D is the LV diameter.
