# Supplementary material for: Weighted–VAE: A deep learning approach for multimodal data generation applied to experimental T. cruzi infection
Source: PLoS One. 2025 Mar 24;20(3):e0315843. doi: 10.1371/journal.pone.0315843 (PMC11932709; doi:10.1371/journal.pone.0315843)
Supplement: S4 Appendix — (PDF) [file pone.0315843.s004.pdf]

Weighted–VAE: A Deep Learning Approach for Multimodal Data Generation Applied to Experimental *T. cruzi* infection

Blanca Vazquez*, Nidiyare Hevia-Montiel, Jorge Perez-Gonzalez, Paulina Haro.
* Corresponding author: blanca.vazquez@iimas.unam.mx

# S4 Appendix: Hyperparameters tuning for multi-classification

We listed the hyperparameters evaluated during the training for multi-classification in Table 1.

**Table 1. Hyperparameters tuning for multi-classification of healthy individuals, and individuals in acute and chronic phase infection.**

| Classifier | Hyperparameters |
|---|---|
| Logistic Regression - LR | C values: log(-3,3,7),<br>Penalty: [elasticnet, l2],<br>Solver:[saga],<br>L1 ratio:[0.5, 0.8] |
| Support Vector Machine - SVM | Kernel: [linear, rbf],<br>Gamma:[auto, scale] |
| Gaussian Process Classifier - GPC | Kernel: [1.0 * rbf(1.0), 1*matern()] |
| Random Forest - RF | Num. estimators: [50,100,200],<br>Max features:[sqrt, log2],<br>Max depth:[2,4,6],<br>Criterion:[gini],<br>Min impurity decrease:[1e−4, 1e−7] |
| Extra Trees Classifier - ETC | Num. estimators: [50,100,200],<br>Max features:[sqrt, log2],<br>Max depth:[2,4,6],<br>Criterion:[gini],<br>Min impurity decrease:[1e−4, 1e−7] |
