# Supplementary material for: Weighted–VAE: A deep learning approach for multimodal data generation applied to experimental T. cruzi infection
Source: PLoS One. 2025 Mar 24;20(3):e0315843. doi: 10.1371/journal.pone.0315843 (PMC11932709; doi:10.1371/journal.pone.0315843)
Supplement: S5 Appendix — (PDF) [file pone.0315843.s005.pdf]

Weighted–VAE: A Deep Learning Approach for Multimodal Data Generation Applied to Experimental *T. cruzi* infection

Blanca Vazquez*, Nidiyare Hevia-Montiel, Jorge Perez-Gonzalez, Paulina Haro.
* Corresponding author: blanca.vazquez@iimas.unam.mx

S5 Appendix: Metrics used to evaluate the performance of multi-classification

The description of the metrics used to evaluated the performance of multi-classification is presented in Table 1.

Table 1. Metrics used to evaluate the performance of multi-classification.

Metric	Description	Formula
Accuracy	Measure the amount of cases classified correctly with respect to the total of cases examined.	Acc = $\frac{TP+TN}{TP+TN+FP+FN}$
F1 score	It is the harmonic mean of precision and recall	F1 = $\frac{2TP}{2TP+FP+FN}$
Recall	It is the ratio of a positive test result, conditioned on the individual truly being positive.	Recall = $\frac{TP}{TP+FN}$
Precision	It is the ratio between the true positives and all the positives.	Precision=$\frac{TP}{TP+FP}$
Normalized Mutual Information	It indicates how much information can be obtained from a random variable by observing another random variable.	$NMI(X,Y) = \sum_{i=1}^{\lvert X\rvert}\sum_{j=1}^{\lvert Y\rvert}\frac{\lvert X_i \cap Y_j\rvert}{N} \log\frac{N\lvert X_i \cap Y_j\rvert}{\lvert X_i\rvert\lvert Y_j\rvert}$

TP true positive; FN false negative; TP true negative; FP false positive; $|X_i|$ is the number of samples of real data X_i, $|Y_i|$ is the number of samples of augmented data Y_i.
