# Supplementary material for: Weighted–VAE: A deep learning approach for multimodal data generation applied to experimental T. cruzi infection
Source: PLoS One. 2025 Mar 24;20(3):e0315843. doi: 10.1371/journal.pone.0315843 (PMC11932709; doi:10.1371/journal.pone.0315843)
Supplement: S8 Appendix — (PDF) [file pone.0315843.s008.pdf]

Weighted–VAE: A Deep Learning Approach for Multimodal Data Generation Applied to Experimental *T. cruzi* infection

Blanca Vazquez*, Nidiyare Hevia-Montiel, Jorge Perez-Gonzalez, Paulina Haro.
* Corresponding author: blanca.vazquez@iimas.unam.mx

S8 Appendix: Performance of multi-classifiers during training in Task 3

Fig 1 and Fig 2 present the results of cross-validation expressed as mean and standard deviation during the training in Task 3.

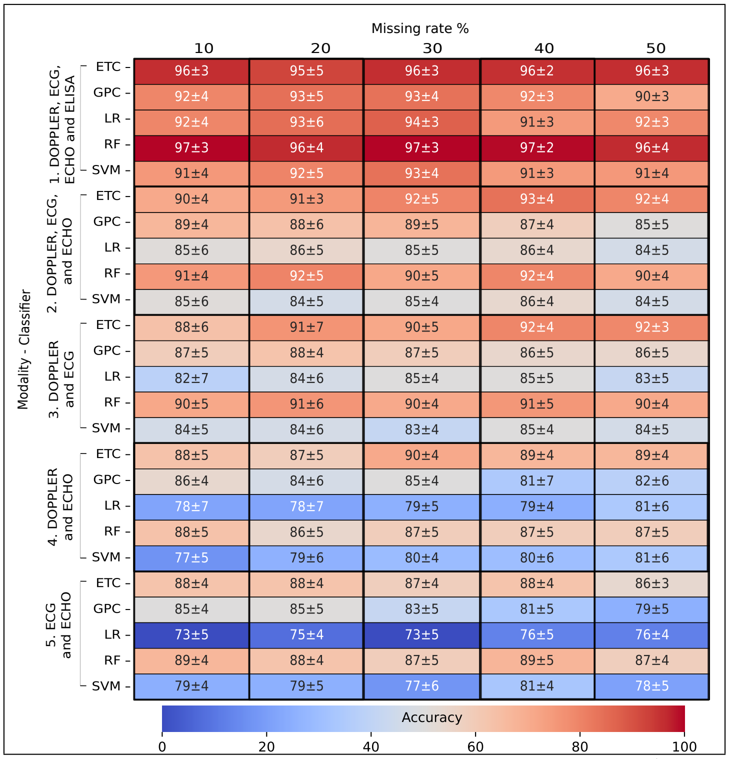

Fig 1. Performance of multi-classifiers during the training (without feature selection) in percentage in Task 3. Each result is shown with mean and standard deviation.

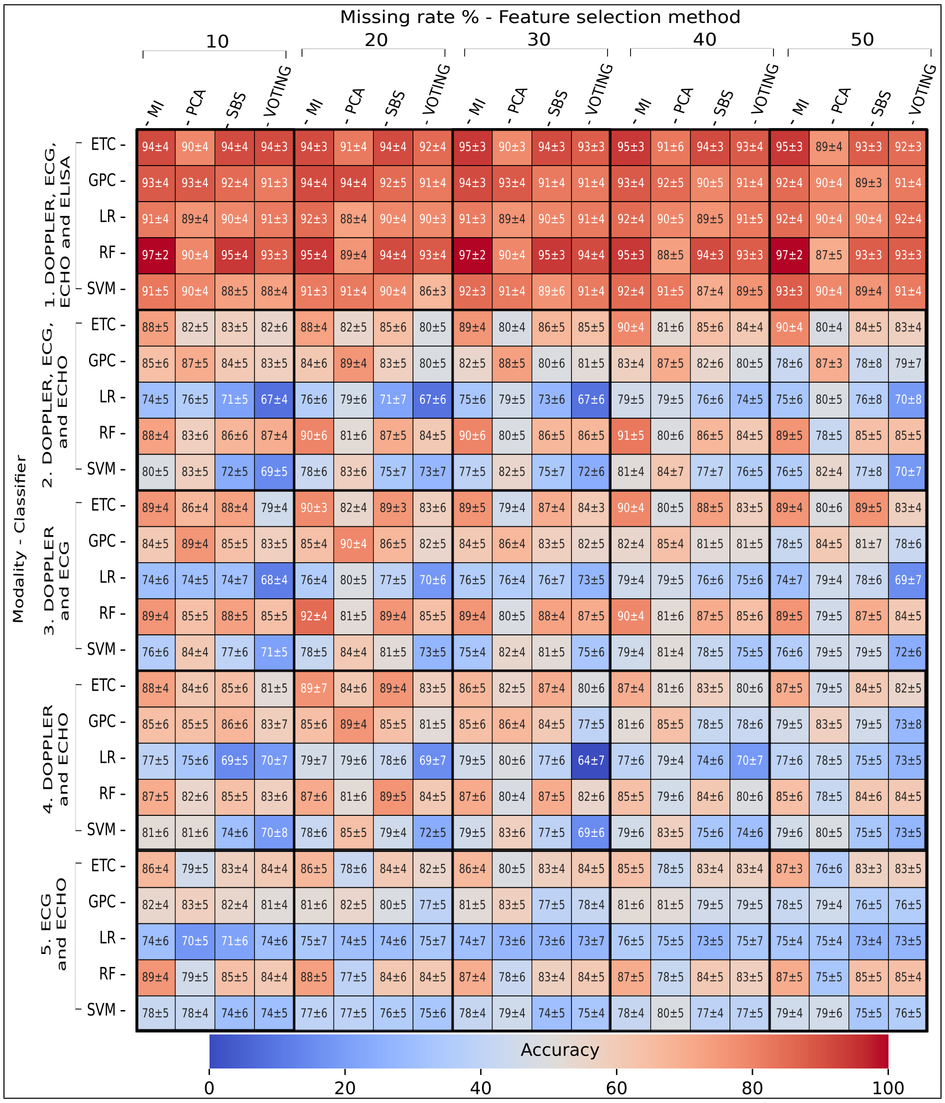

Fig 2. Performance of multi-classifiers during the training (with feature selection) in Task 3. Each performance is shown with mean and standard deviation.
