# Supplementary material for: Weighted–VAE: A deep learning approach for multimodal data generation applied to experimental T. cruzi infection
Source: PLoS One. 2025 Mar 24;20(3):e0315843. doi: 10.1371/journal.pone.0315843 (PMC11932709; doi:10.1371/journal.pone.0315843)
Supplement: S9 Appendix — (PDF) [file pone.0315843.s009.pdf]

Weighted–VAE: A Deep Learning Approach for Multimodal Data Generation Applied to Experimental *T. cruzi* infection

Blanca Vazquez*, Nidiyare Hevia-Montiel, Jorge Perez-Gonzalez, Paulina Haro.
* Corresponding author: blanca.vazquez@iimas.unam.mx

S9 Appendix: Analysis of probability density function

We presented the probability density function of the real and generated data by class and by modality on test set. In each figure, the row presents a feature and each column shows the density of this feature at level: 1) overall, 2) healthy individuals, 3) acute phase individuals, and 4) chronic phase individuals.

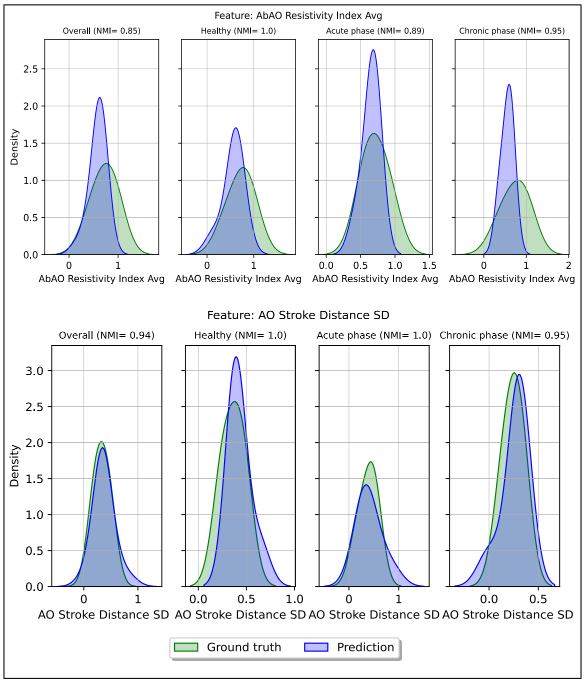

Fig 1. DOPPLER modality: comparison between a kernel density estimate between real and generated data by the proposed W–VAE. Each row presents a feature and each column shows the density of this feature: 1) overall, 2) healthy individuals, 3) acute phase individuals, and 4) chronic phase individuals.

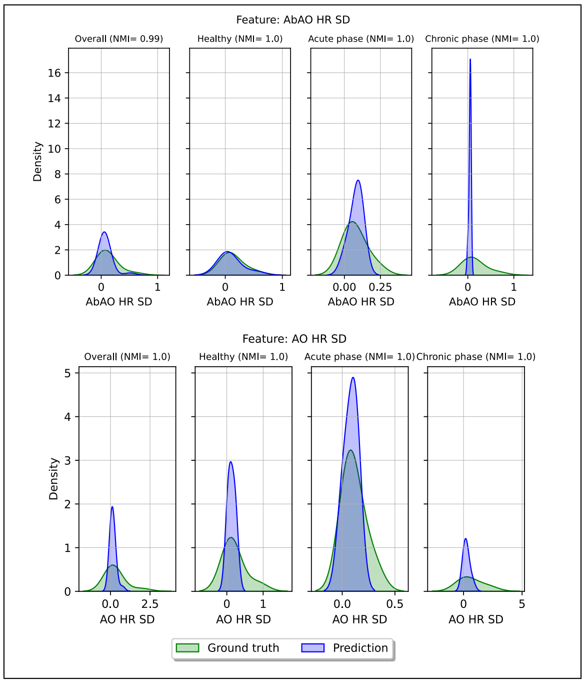

Fig 2. DOPPLER modality (continued): Comparison between a kernel density estimate between real and generated data by the proposed W–VAE.

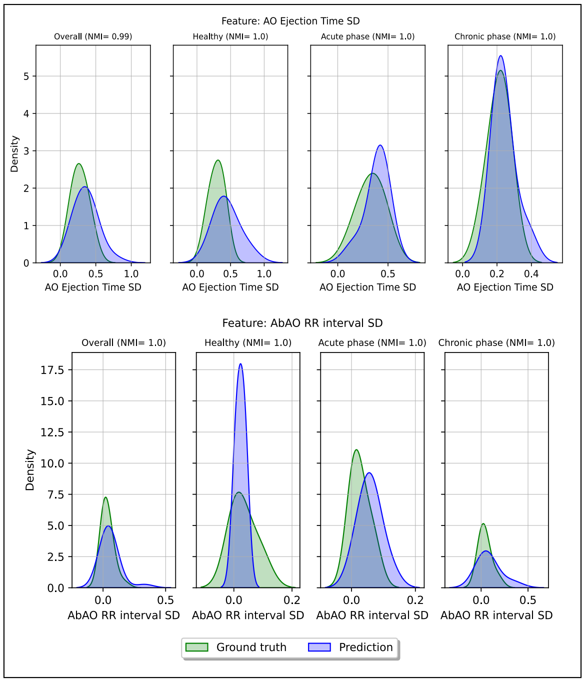

Fig 3. DOPPLER modality (continued): Comparison between a kernel density estimate between real and generated data by the proposed W–VAE.

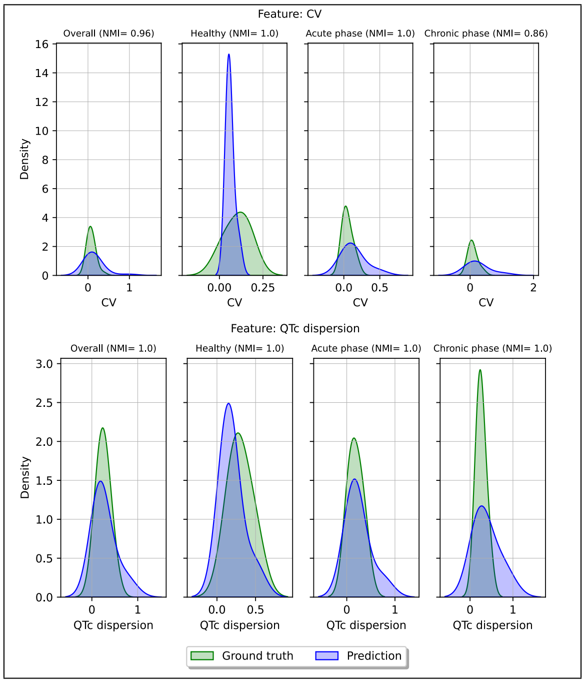

Fig 4. ECG modality: Comparison between a kernel density estimate between real and generated data by the proposed W–VAE.

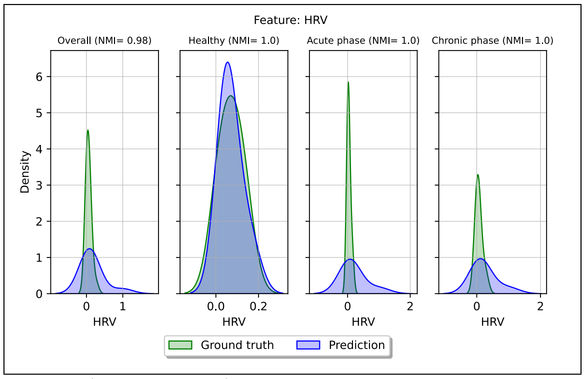

Fig 5. ECG modality (continued): Comparison between a kernel density estimate between real and generated data by the proposed W–VAE.

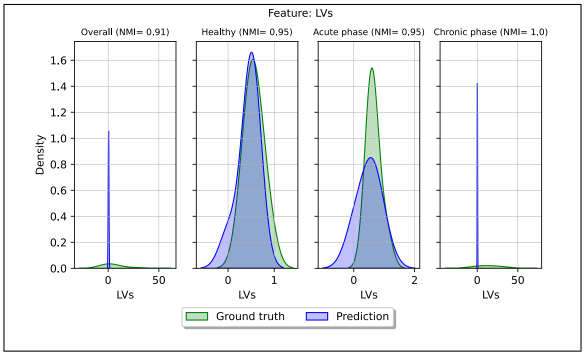

Fig 6. ECHO modality: Comparison between a kernel density estimate between real and generated data by the proposed W–VAE.

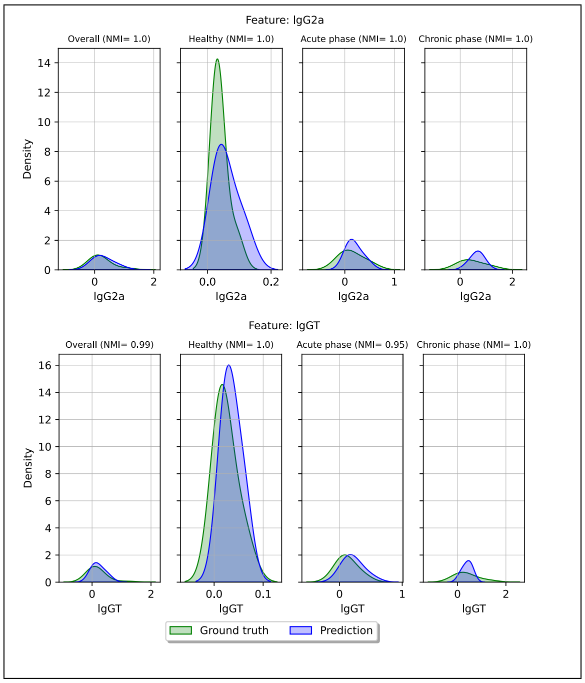

Fig 7. ELISA modality: Comparison between a kernel density estimate between real and generated data by the proposed W–VAE.

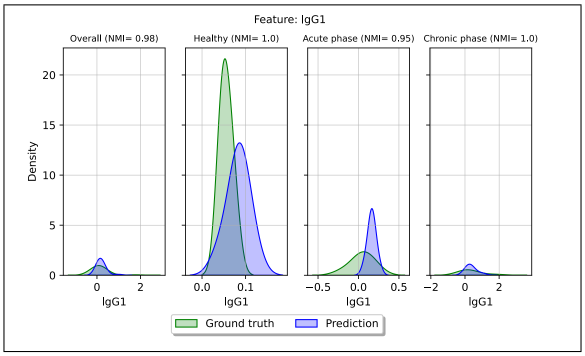

Fig 8. ELISA modality (continued): Comparison between a kernel density estimate between real and generated data by the proposed W–VAE.
